# Assembly and analysis of Sinipercidae fish sex chromosomes reveals that a supergene drives sex chromosome origin and turnover

**DOI:** 10.1007/s44307-025-00068-6

**Published:** 2025-05-28

**Authors:** Chong Han, Shiyan Liu, Suhan Peng, Shuang Liu, Junyan Zeng, Jiehu Chen, Haoran Lin, Cai Li, Shuisheng Li, Yong Zhang

**Affiliations:** 1 State Key Laboratory of Biocontrol and School of Life Sciences, Southern Marine Science and Engineering Guangdong Laboratory (Zhuhai), Guangdong Provincial Key Laboratory for Aquatic Economic Animals and Guangdong Provincial Engineering Technology Research Center for Healthy Breeding of Important Economic Fish, Institute of Aquatic Economic Animals, Sun Yat-Sen University, Guangzhou, 510275 China; 2 Laboratory for Marine Fisheries Science and Food Production Processes, Qingdao Marine Science and Technology Center, Qingdao, Shandong 266237 China; 3 School of Life Sciences, Guangzhou University, Guangzhou, 51006 China; 4 Science Corporation of Gene (SCGene), Guangzhou, 510080 China

**Keywords:** Mandarin fish, Sex chromosome assembly, *Amhy*, Sex-determining gene, Origin and turnover of sex chromosome

## Abstract

The sex chromosomes of fish are usually homomorphic and recently derived, making them an ideal model to understand the origin and evolution of sex chromosomes in vertebrates. Here, combined Pacbio, Hi-C, and Illumina sequencing of the male mandarin fish (*Siniperca chuatsi*) genome enabled the assembly of a high-quality chromosome-level genome, including highly resolved X and Y chromosome assemblies. Genome wide association study (GWAS) and coverage information analysis revealed a 2.0 Mbp sex determining region (SDR). A Y-specific duplication of the anti-Müllerian hormone (*amh*) gene, *amhy*, was identified that encoded a truncated AMH protein. Loss of function and overexpression experiments demonstrated that *amhy* may act as a male sex-determining gene (SDG). GWAS led to the identification of a common sex chromosome (Chr24) and SDG (*amhy*) in *S. scherzeri*, while another sex chromosome (Chr11) was identified in *Coreoperca whiteheadi*. Interestingly, completely conserved protein coding sequences of *amhy* were commonly identified in male *S. chuatsi*, *S. scherzeri,* and *S. knerii*. These results support a classic model for the origin and evolution of early sex chromosomes and suggest that *amhy* is a super SDG that can drive the origin and turnover of sex chromosome in vertebrates.

**Supplementary Information:**

The online version contains supplementary material available at 10.1007/s44307-025-00068-6.

## Introduction

The origin and evolution of sex chromosomes have remained intense, but unresolved areas of research. Sex chromosome pairs are known to have evolved from a pair of autosomes (Bachtrog 2013). After divergence of alleles or gene replication and translocation, autosomes obtain sex determining regions (SDRs). Recombination is then suppressed, while mutations and repetitive DNA further accumulate in areas adjacent to SDRs, further enlarging SDRs and forming heteromorphic sex chromosomes (Schartl et al. 2016). Traditional perspectives have suggested that heteromorphic sex chromosomes are an “evolutionary trap”. However, recent studies have revealed that sex chromosome sequences are much more dynamic and are often accompanied by loss or gain of genes (Bellott et al. 2017; Soh et al. 2014). Complexity involved in the origin and evolution of sex chromosomes in vertebrates has led to considerable uncertainty in these processes. Sex chromosomes have differentiated for a long time in birds and mammals, wherein Y or W chromosomes are highly heteromorphic and contain poor genes and abundant repetitive sequences (Bellott et al. 2014; Xu et al. 2019). In contrast, fish are lower vertebrates and have the most diverse sex determination systems, encompassing almost all sex determination systems found in vertebrates. Concomitantly, their sex chromosomes are often homomorphic and recently derived (Li and Gui 2018). Thus, fish are ideal subjects to investigate the origin and evolution of vertebrate sex chromosomes.

The rapid development of single-module sequencing of long reads (i.e., Pacbio sequencing) and chromatin conformation capture sequencing (Hi-C) technology has led to increasing numbers of assembled fish sex chromosomes, including for the three-stickleback (*Gasterosteus aculeatus*) (Peichel et al. 2020), Greenland halibut (*Reinhardtius hippoglossoides*) (Ferchaud et al. 2022), spotted knifejaw (*Oplegnathus punctatus*) (Li et al. 2021), and zig-zag eel (*Mastacembelus armatus*) (Xue et al. 2021), among others. The mechanism of sex determination has been identified in many fish species. However, the diversity of sex determination mechanisms in fish renders it difficult to understand the origin and evolution of sex chromosomes. Sex determination systems are highly conserved in mammals and birds, but are variable in fish, even in different species within the same genus like *Oryzias* (*Oryzias hubbsi*: ZW, *Oryzias dancena*: XY) (Graves and Peichel 2010; Myosho et al. 2012; Takehana et al. 2014, 2007) and *Oreochromis* (*Oreochromis niloticus*: XY, *Oreochromis aureus*: ZW) (Tao et al. 2021). A previous study identified signatures of sex chromosomes in 79 cichlid taxa, demonstrating that about 12 chromosomes were separately selected as sex chromosomes in different species, supporting the “limited option” hypothesis for sex chromosome evolution in vertebrates. However, no association was observed between the size of a reference linkage group (LG), the number of genes, or the number of known SD candidate genes on a reference LG with the frequency by which these LGs became a sex chromosome in cichlids (El Taher et al. 2021). As more taxa are investigated, additional complexities inconsistent with existing theory will likely arise (Abbott et al. 2017). Thus, investigating closely related species may be a more insightful means to explore sex chromosome origin and evolution (Pan et al. 2019; Ross et al. 2009).

Mandarin fish within the Perciformes and Sinipercidae taxa include 2 genera and 12 species. The species are economically important freshwater fish that is primarily distributed in rivers of East and Southeast Asia including China, Russia, Vietnam, Korea and so on (He et al. 2020). These species have been regarded as a valuable freshwater product for as long as thousands of years in China (Han et al. 2020). The total aquaculture production of mandarin fish cultured in China reached 477,592 tons in 2023. Among them, *Siniperca chuatsi* has the fastest growth rate, so it has been the main type of cultured mandarin fish in China. Moreover, female mandarin fish (*S. chuatsi*) exhibit better growth performance (by 10–20% of body weight) than males (Sun et al. 2017). Thus, all-female mandarin fish breeding can effectively increase aquaculture production. Although chromosome-level genome assemblies of *S. chuatsi* have been generated (He et al. 2020; Yang et al. 2022), they were derived from female fish and sex chromosomes were not identified. Later studies identified that *S. chuatsi* exhibited a heteromorphic XY sex chromosome system that is shared across the *Siniperca* genus (Han et al. 2020; Wen et al. 2022). Moreover, an approximately 5.0 Mbp SDR was identified in the candidate sex chromosome LG2 of *S. chuatsi* using illumina pool sequencing, although no sex determining gene (SDG) was identified in this region (Wen et al. 2022). Most studies of mandarin fish sex determination have relied on short-read sequencing data that does not accurately span highly repetitive regions in sex chromosomes. The lack of sex chromosome assembly for mandarin fish has continued to limit our understanding of their sex determination and sex chromosome evolution. In addition, the white head mandarin fish (*Coreoperca whiteheadi*) is a proximal species in the genus *Coreoperca* that is considerably differentiated from species in the genus *Siniperca*, with its SDR being unique (Wen et al. 2022). Thus, sex chromosome evolution appears to have occurred within Sinipercidae fishes.

To characterize the origin and evolution of sex chromosomes in the Sinipercidae, a high-quality chromosome-level assembly was generated here for *S. chuatsi,* alongside the first assembled sex chromosomes based on Pacbio, high-throughput chromatin conformation capture (Hi-C), and Illumina sequencing. A subsequent genome wide association study (GWAS) identified an approximately 2.0 Mbp SDR that contained a candidate SDG, *amhy*. Gene knockout and overexpression of *amhy* demonstrated that it may determine the male sex of *S. chuatsi*. Further, genomic structure and dual luciferase reporter assays revealed that *amhy* evolved rapidly and can inhibit the expression of *Cyp19a1a* in multiple different ways. Comparison of the X and Y chromosomes also revealed the presence of SDG duplication and translocation, recombination inhibition, and repeat sequence accumulation that occurred in the SDR. Further gene comparison and GWAS investigations revealed that the highly conserved *amhy* drove the turnover of sex chromosomes in the genus *Siniperca*. Overall, these results document a prototypical example of an SDG-driven origin and turnover of early sex chromosomes in vertebrates.

## Results

### Chromosome-level genome assembly and identification of sex chromosomes

**Fig. 1:**
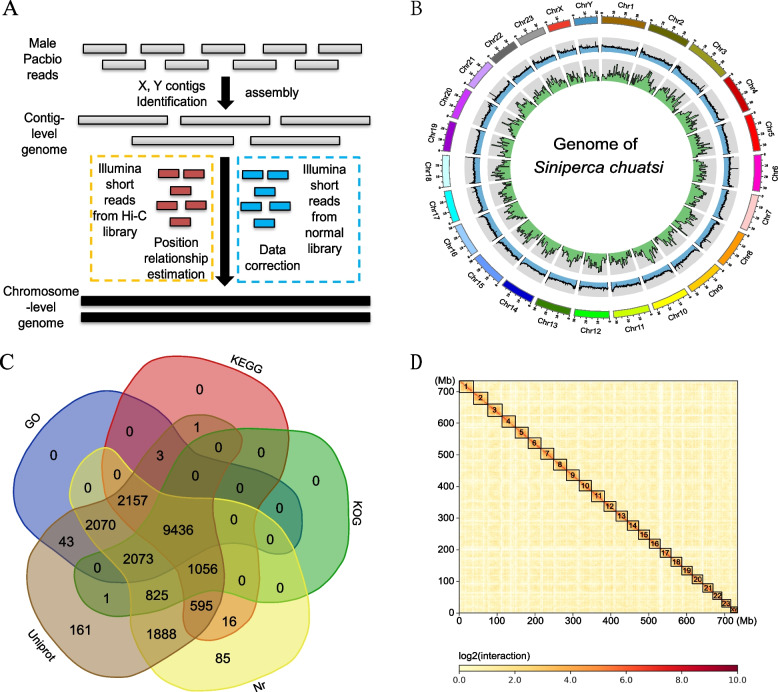
Characteristics of the chromosome-level genome assembly of male *S. chuatsi*. ***A*** Workflow for the chromosome-level assembly. ***B*** Circos map of the male genome. The first circle indicates chromosome length, the second circle indicates the distribution of GC proportion, with a statistical window of 100 kbp and without overlap. The third circle indicates the distribution of gene density over a window of 1 Mbp and without overlap. ***C*** Venn diagram of the number of gene annotations based on comparisons against five databases. ***D*** Genome Hi-C interaction diagram. The density of interactions is showed by the color bar on the right

The sizes of the female and male *S. chuatsi* genomes were previously estimated to be 973 and 972 Mbp, respectively, based on Illumina sequencing (Han et al. 2020). One adult XY male *S. chuatsi* with high genetic homozygosity was selected for genome sequencing and assembly. A high-quality genome assembly was generated for the XY male using a combination of PacBio long read, Illumina short read, and Hi-C sequencing technologies (Fig. 1A; Supplemental Table S1). In addition, Y-link contigs were identified according to sequencing depth and previously identified male-specific marker sequences. Further, X-link contigs were identified according to shared sequence homology to Y-link contigs and sequencing depth (~ 0.3 to 0.7 times the overall genomic depth) (Han et al. 2021). The haplotype genome of the XY male was 750.13 Mbp and comprised 24 chromosomes (N50 = 31.40 Mb), with the longest chromosome length being 39.54 Mbp (Chr3). The lengths of the X and Y chromosomes were 19.98 (43 contigs) and 20.46 (42 contigs) Mbp, respectively, representing the shortest chromosome pair (Fig. 1B; Supplemental Table S2). Iso-seq analysis of multiple tissues (71.68 Gbp) and Illumina RNA-seq of gonads (133.25 Gbp) revealed a total of 25,122 protein coding genes that were annotated (Fig. 1C). BUSCO analysis suggested that 98.68% and 98.43% of the 3,650 BUSCO groups were complete in the genome comprising sequences that were not assembled at the chromosome level and those assembled at the chromosome level, respectively (Supplemental Table S3). These completeness levels were higher than in previous studies (He et al. 2020; Yang et al. 2022). Further, Hi-C sequencing revealed continuous and strong positional relationships of super scaffolds within the chromosomal groups (Fig. 1D).

The alignment of the X and Y chromosomes revealed that nearly 90% of the X and Y chromosomes were completely conserved and only the initial ~ 2.0 Mbp (X-link region) of X chromosomes (6 contigs) and 2.38 Mbp (Y-link region) of Y chromosomes (5 contigs) exhibited little difference (Supplemental Fig. S1 A). Even within this region, gene compositional analysis revealed that most genes were shared between the X (125 genes) and Y (112 genes) chromosomes, with only a few different genes that differed between them (10 Y-specific and 28 X-specific genes; Supplemental Table S4). One gene that was different between the two was annotated as the anti-mullerian hormone (*amh*) gene and was only present in the Y-link region. Gene synteny analysis also interestingly revealed that only genes around *amhy* were clearly different, with even some gene replication and gene inversion events being observed (Supplemental Fig. S1B and S1 C). The pseudogene distribution of all chromosomes was also estimated, revealing an extremely high ratio of pseudogenes in the X and Y-link regions (Supplemental Table S5). Moreover, repetitive elements exhibited little difference between the X and Y chromosomes and were primarily enriched near the centromeres of the sex chromosomes (especially Simple repeats and DNA transposons) (Supplemental Fig. S1D; Supplemental Fig. S2).

### Identification and characterization of SDRs

**Fig. 2:**
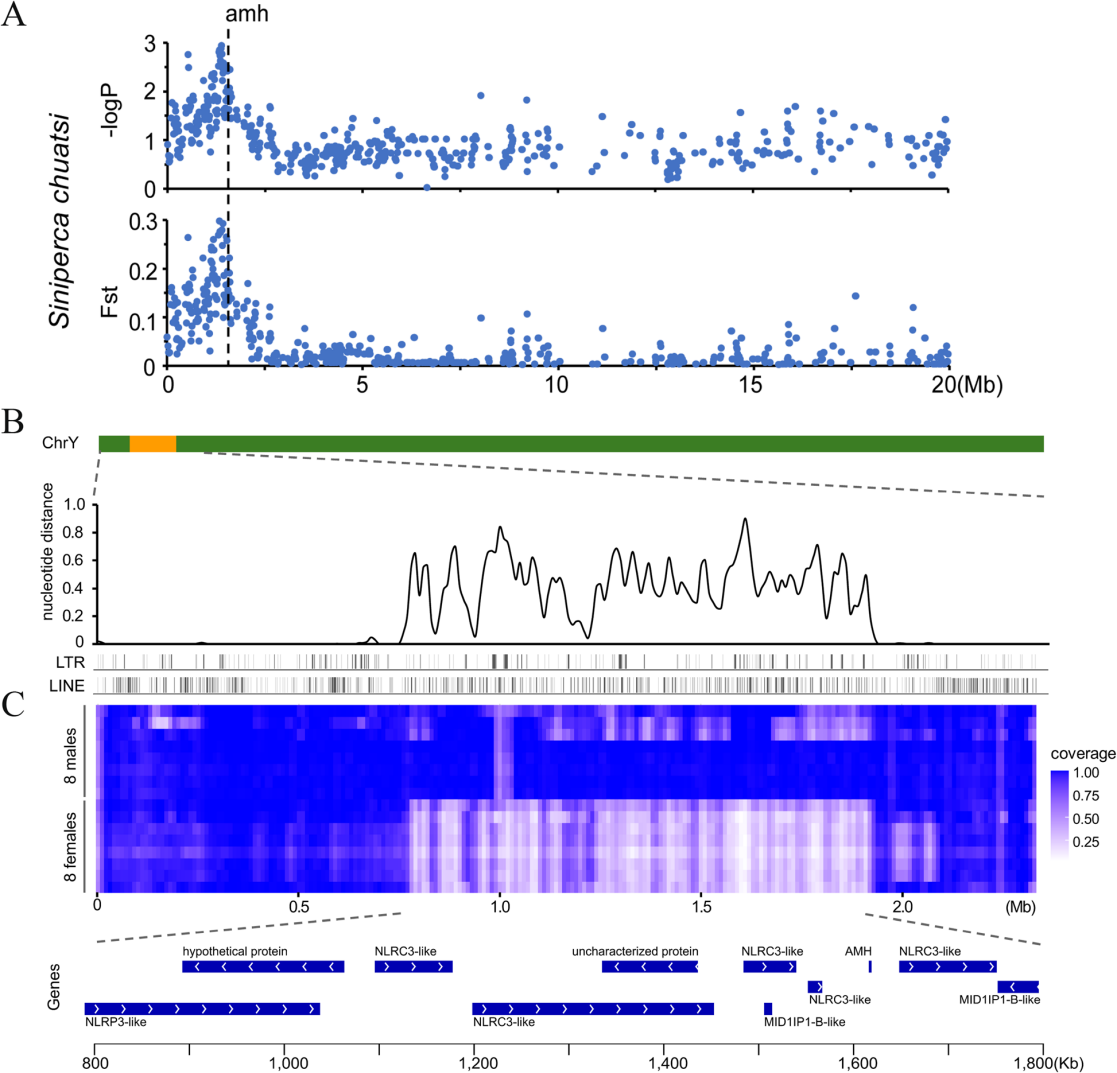
Characterization of the SDR in the Y chromosome of *S. chuatsi*. ***A*** Manhattan plot showing the relationship between the –logP of sex-related SNPs and Fst values for males (*n* = 8) and females (*n* = 8) across ChrX. The *Amh* location is indicated by a vertical dotted line. The male-specific region was not included in the analysis, since it is missing on ChrX (The *P*-value in GWAS analysis represents the significance of the association between genotype and phenotype. The smaller the *P*-value (or the larger the -logP value), the more significant the association is). ***B*** Nucleotide distance and repetitive elements (LTR and LINE) of the SDR over 50 kbp windows, with steps of 25 kbp. ***C*** Coverage of sequenced individuals across the SDR over 50 kbp windows, with steps of 25 kbp. Regions with high coverage are indicated with blue blocks. The oriention (white arrow) and location (blue bar) of candidate genes in 0.8–1.8 Mb are revealed at the bottom

To further characterize the SDR, whole genome sequence data from eight XY males and eight XX females were mapped to the assembled genome of *S. chuatsi*. A total of 4,776,557 SNP loci were detected, with 15,885 SNPs located in the Y-link region (Supplemental Table S6). Statistical analysis revealed that sex specific SNPs were significantly enriched in the 0.5–1.9 Mbp regions (-logP > 2), especially in the region near *amhy* (Fig. 2A, Supplemental Fig. S3). The nucleotide distances between the X and Y chromosomes in the sex-associated region were calculated, with regions exhibiting nucleotide distances > 0 primarily centered in the range of 0.8–1.8 Mbp (Fig. 2B). Low coverage was also found in this region for all female individuals (Fig. 2C). Moreover, maximal nucleotide distances and nearly absent coverage for females were identified in the *amhy* region. Thus, these results suggested that the candidate SDR region was primarily located in the 0.5–1.9 Mbp area, and especially in the 0.8–1.8 Mbp region. In addition, *amhy* was implicated as a candidate SDG for *S. chuatsi*.

Transcriptome and cloning analysis via RACE revealed only one coding isoform that corresponded to exon 1, 5–7 of the autosomal paralog *amh* was found in *amhy* (Supplemental Fig. S4 A), while the important AMH and TGFβ domains were retained (Supplemental Fig. S4B and S4 C). Sequence alignment of *amhy* with its paralog *amh* on chromosome 8 demonstrated that *amhy* encodes a severely truncated form of *amh* (Supplemental Fig. S4D). Two-hundred and forty individuals with known sex were further subjected to genotype analysis to identify the presence of *amhy*. Surprisingly, *amhy* was present in all males, but not in any females, suggesting a strong association with sex determination (Supplemental Table S7).

In addition to the above, many NOD-like receptor family CARD domain containing 3 (*nlrc3*) genes were identified in the SDR, with 16 on the X chromosome and 23 on the Y chromosome. Evaluation of *nlrc3* at the genome-wide level revealed that *nlrc3* was specifically enriched in the SDR. Moreover, an additional 6 NACHT, LRR, and PYD domain-containing protein 3 (*nlrp3*) genes were identified on the X chromosome and 9 on the Y chromosome. Other Y-specific genes were identified in this region, including those encoding histone-lysine N-methyltransferase, CD3 zeta, chromosome alignment-maintaining phosphoprotein 1, enterin neuropeptide, melanophilin, and protein EFR3 homolog B (Supplemental Table S4). Thus, gene tandem replication and gene insertion likely occurred in the SDR.

Abundant repetitive elements were also enriched in the SDR, including DNA transposons (3.34% and 4.84% of the region sequence, respectively), long terminal repeat retrotransposons (LTRs, 0.98% and 0.96%, respectively), and long interspersed elements (LINEs, 4.87% and 5.64%) (Supplemental Table S8). Among these, several subfamilies of LINEs (L2/CR1/Rex, RTE/Bov-B), LTRs (Gypsy/DIRS1) and DNA transposons (Tc1-IS630-Pogo) clearly accumulated in the X-link and Y-link regions. The ratio of hobo-activator and Tourist/Harbinger types of DNA transposons in the X-link region were significantly higher than in the Y-link region, while the R1/LOA/Jockey and L1/CIN4 types of LINEs and retroviral LTRs accounted for higher proportions in the Y-link region. Considering both the whole sex chromosome and the SDG region of sex chromosomes, the ratio of repeat sequences on the Y chromosome was higher than for the X chromosome.

### *Amhy* is necessary for male sex determination

**Fig. 3:**
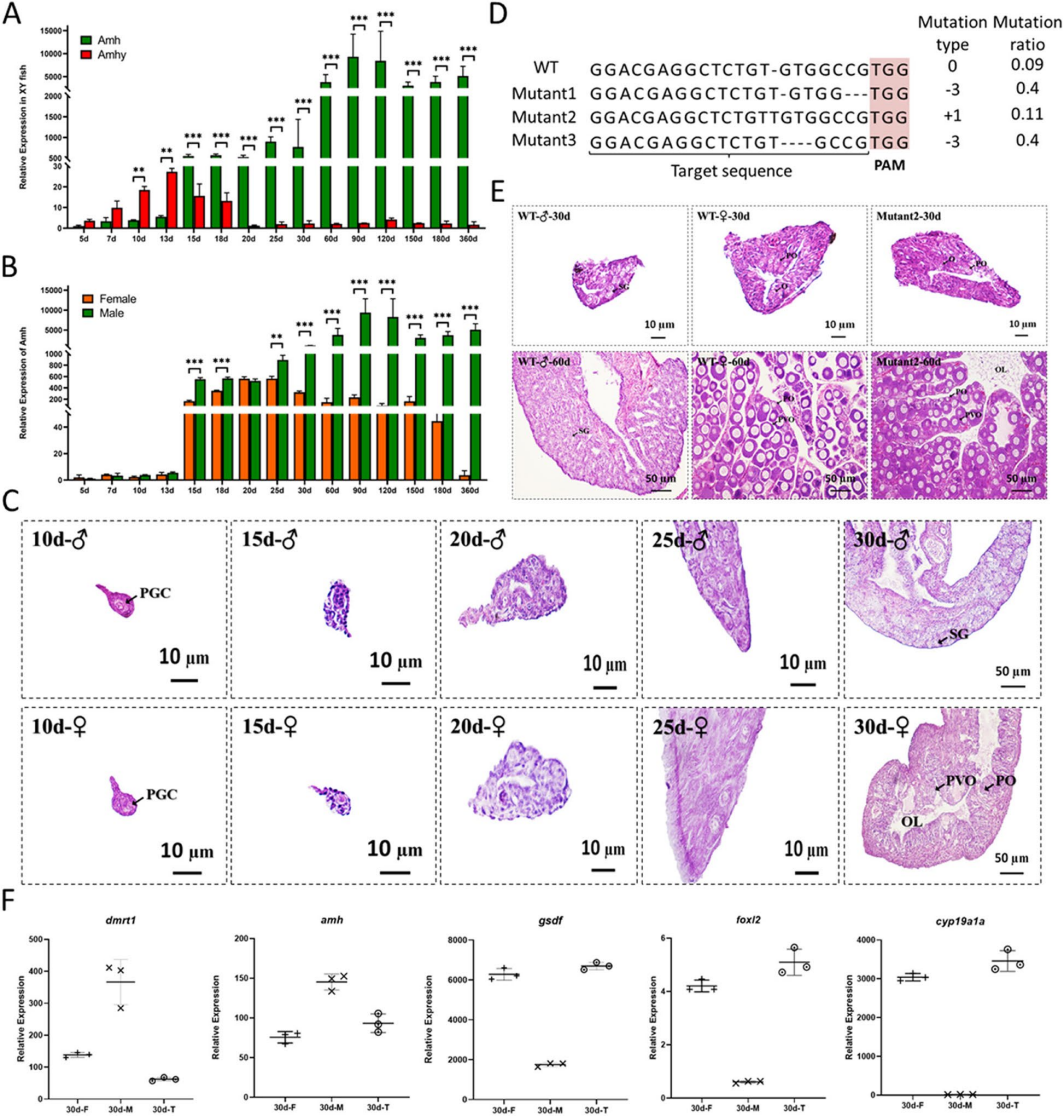
Expression and functional analysis of *amhy* in *S. chuatsi*. ***A*** Expression analysis of *amh* and *amhy* in male gonads at 5–360 dah based on RT qPCR. ***B*** Expression analysis of *amh* in female and male gonads at 5–360 dah based on RT qPCR. ***C*** Gonadal histology of male and female mandarin fish at 10–30 dah. G: Gonium; O: oogonium; OL, ovarian lumen; SG: spermatogonia; PVO, pre-vitellogenic oocytes. ***D*** Mutations and mutation rate in the F1 generation of *amhy* knockouts (WT: wild type). ***E*** Gonadal histological analysis of *amhy*-knockout individuals (Mutation2), WT males, and WT females at 30 and 60 dah. ***F*** Transcriptional expression of male (*dmrt1*, *amh* and *gsdf*) and female marker genes (*foxl2* and *cyp19a1a*) in gonad tissues at 30 dah based on *amhy* knockout individuals (30 d-T), WT females (30 d-F), and WT males (30 d-M)

Early gonad transcriptome data suggested that many sex determination and differentiation genes exhibited obvious differential expression at 15 days after hatching (dah) (Supplemental Fig. S5). The expression of female-biased genes like *foxl2* and *cyp19a1a* gradually increased in the female gonad 15 dah, but decreased gradually in male gonads. The expression of male-biased genes like *dmrt1*, *gsdf,* and *amh*, rapidly increased in male gonads 15 dah (Supplemental Fig. S5), as confirmed with RT qPCR (Supplemental Fig. S6). Tissue and gonad expression analysis of *amhy* and the autosomal copy *amh* were conducted. In different tissues of adult male mandarin fish, *amhy* expression was only observed in gonads, while low *amh* expression was detected in most tissues, but with only relatively high expression levels in gonads, and especially the testis (Supplemental Fig. S7). Interestingly, *amhy* was primarily expressed in testis 7–18 dah, peaked at 13 dah, and only exhibited weak expression at 20–360 dah, while *amh* expression in the testis was less than that of *amhy* 5–15 dah, but higher than that of *amhy* 15–360 dah (Fig. 3A). *amh* expression significantly increased in female and male gonads 15 dah, but expression in male gonads was significantly higher than in female gonads (Fig. 3B). Alignment of the promoter sequences of *amh* and *amhy* revealed large differences (nucleotide identity = 39.45%) (Supplemental Fig. S8) and there were many predicted transcription factor binding sites located on *amhy* promoter but not on *amh* promoter (Supplemental Table S9), which could explain their different expression patterns. Gonad histology was also evaluated during the early stage of differentiation. Primordial germ cells (PGC) appeared in gonads 10 dah and cell morphology differentiated between female and male gonads at about 20 dah. However, obvious spermatogonium (SG) and oogonium (O) appeared in gonads 30 dah (Fig. 3C). Overall, these results suggest that a critical time point for male and female gonad differentiation at the level of gene expression is 15 dah and that *amhy* may initiate the divergence of gene expression between male and female mandarin fish.

To further investigate the function of *amhy* in male sex determination, CRISPR/Cas9 technology was used to knock out *amhy* in *S. chuatsi*. One target in the first exon was effective for inducing deletions in *amhy* (Supplemental Fig. S9 A) using the T7 Endonuclease I (Supplemental Fig. S9B), as confirmed by sequencing (Supplemental Fig. S9 C). Moreover, a nearly 74% mutation rate and about six types of frameshift mutations were caused by this target (Supplemental Fig. S9D). F0 generation mutants were further mated with normal females to obtain F1 generation individuals. Sequence analysis of F1 generation male individuals revealed only three types of mutations in F1 generation genetic male individuals, among which only Mutant2 (11%) was a frameshift mutation that exhibited one more base than the wild type (WT). Mutant2 was used as the *amhy*-knockout for *S. chuatsi* (Fig. 3D). At 30 and 60 dah, mutant2 gonads were analyzed via histological sections, with the histology revealing ovary-like morphologies containing ovogonium (O) and primary oocytes (PO) 30 dah and complete ovarian morphology filled with pre-vitellogenic oocytes (PVO) 60 dah (Fig. 3E). Gonad gene expression analysis of Mutant2 at 30 dah revealed that male (*dmrt1*, *amh* and *gsdf*) and female marker genes (*foxl2* and *cyp19a1a*) of *amhy*-knockout fish were similar to those of WT females (Fig. 3F). An *amhy* overexpression plasmid was also constructed and then fed to the all-female juvenile mandarin fish (15 dah) after embedding in lipidosomes. Gonad histology analysis at 60 and 90 dah revealed that gonads developed into testis in the *amhy* overexpression group (Supplemental Fig. S10). Overall, these results demonstrated that *amhy* was the male SDG in mandarin fish.

### Accelerated protein evolution and physiological activity of AMHY

**Fig. 4:**
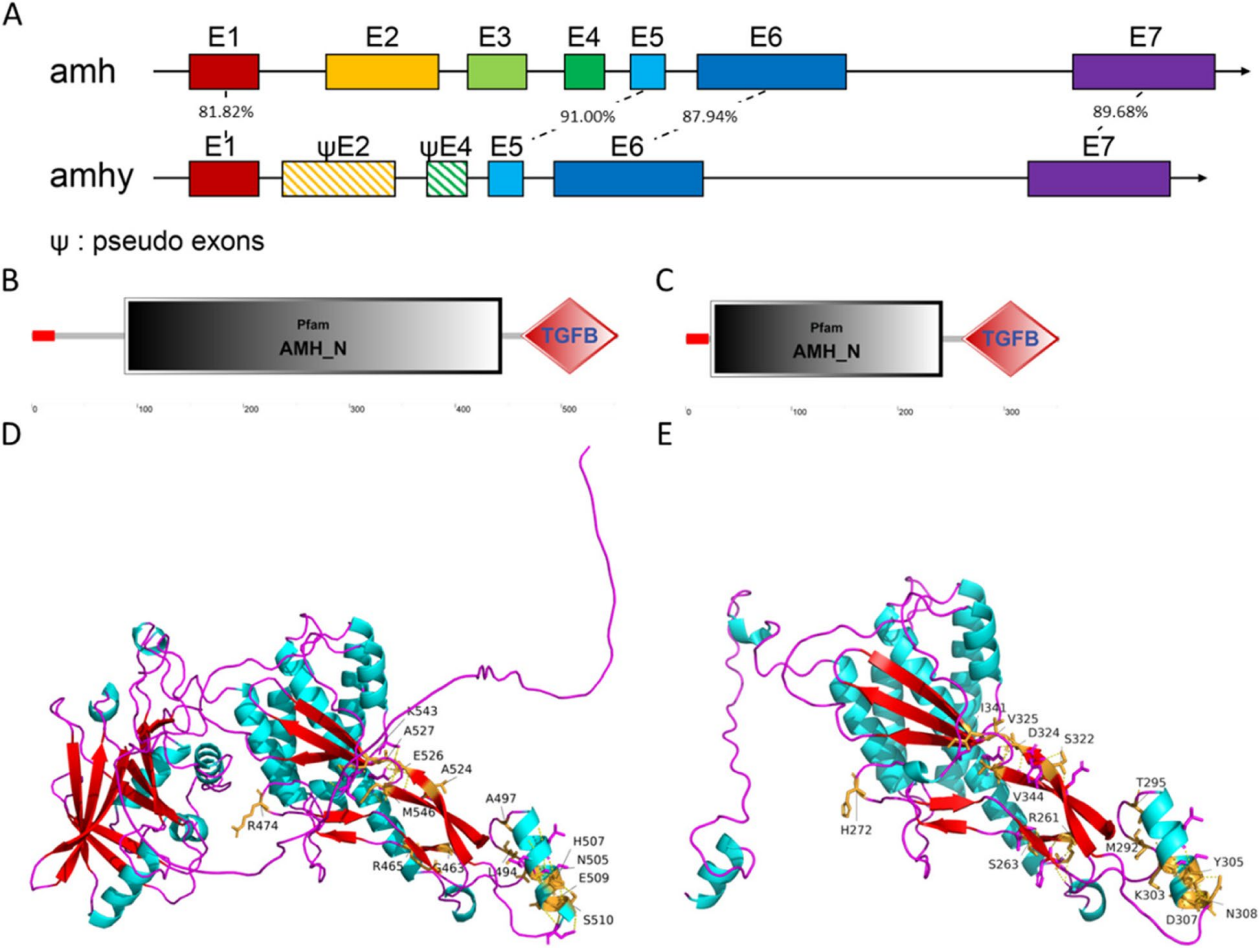
Structural analysis of *amh* and *amhy*. ***A*** Gene structure and exon identity of *amh* and *amhy* sequences. ***B*** Domain composition of *amh*, where red indicates the signal peptide. ***C*** Domain composition of *amhy*. ***D*** Crystal structure of the AMH and TGFβ domains of AMH. Yellow colors indicate amino acids that differ in the domains. ***E*** Crystal structure of the AMH and TGFβ domains of AMHY. Yellow colors indicate amino acids that differ in the domains

There were large fragment deletions in the front region of the coding sequence (CDS) of *amhy* compared with the *amh* gene. Even in homologous regions, many base differences were observed and the overall CDS similarity was only 52.52% (amino acid identity of 50.90%) (Supplemental Fig. S4D; Supplemental Table S10). Sequence identity was significantly lower than other species harboring duplicate *amh* (Supplemental Table S10). Genome sequence alignment of *amhy* and *amh* revealed that *amhy* comprised 4 exons, while *amh* comprised 7 exons. In addition, traces of *amh* pseudo exons including exon2 and exon4 were also detected in the first intron of *amhy*, with no trace of exon3 (Fig. 4A). Despite large differences, structural prediction indicated that *amhy* encoded the complete TGFβ and AMH domains, but the AMH domain of AMHY was significantly shorter than that of autosomal AMH (Fig. 4B and C). Three-dimensional protein structural analysis indicated a large deletion of the amino acid fragment in the N terminal of AMHY, while the TGF domains of AMH and AMHY were highly similar, with only 14 amino acid differences (Fig. 4D and E). Ka/Ks analysis of *amhy*/*amh* also revealed that *amhy* underwent relaxed purifying selection, while only exons 1 and 5 of *S. chuatsi* underwent positive selection (Supplemental Fig. S11). Analysis of Ks values suggested that the duplication of *amh* occurred between ~ 44 and ~ 51 million years ago (Supplemental Table S11). Thus, despite that AMHY exhibits accelerated protein evolution, the mutant form of AMHY still retains AMH function.

**Fig. 5:**
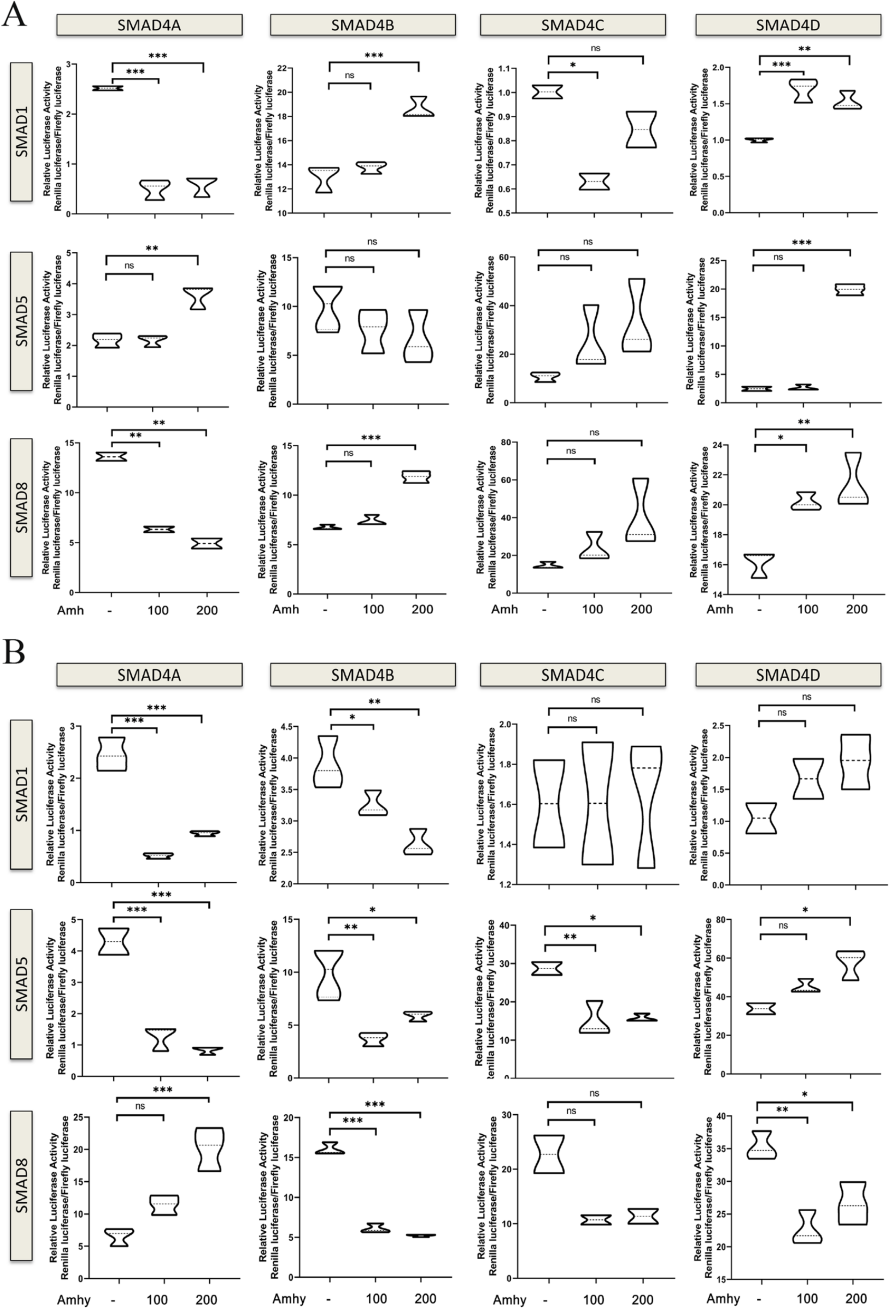
Differential activation of *cyp19a1a* promoter upon selective *amh* (***A***) of *amhy* (***B***) expression by a luciferase reporter assay in HEK 293 T cell co-transfected with different combinations of SMAD1/5/8 and SMAD4 A/4B/4 C/4D

Many studies have demonstrated that *amh* has a negative inhibitory effect on the expression of aromatase *cyp19a1a* via the Amhr2/SMADs signaling pathway (Sacchi et al. 2016; Liu et al. 2021). Based on Alpha Fold 3 server, the protein interaction sites between AMH and AMHRII, AMHY and AMHRII were successfully predicted (Supplementary Supplemental Fig. S12 and S13; Supplemental Table S12). The results showed that interaction sites located in the extracelluar domain of AMHRII were similar between AMH and AMHY, with only three different sites. However, the interaction sites located in TGFβ domain of AMH and AMHY were extremely different. To investigate the activity of AMHY, the promoter of *cyp19a1a* was cloned along with the CDS of *amhr2* and all smad genes of *S. chuatsi*. The effect of AMHY on the activity of the *cyp19a1a* promoter was then investigated with different combinations of SMADs. AMHY significantly inhibited the activity of the *cyp19a1a* promoter via multiple combinations including SMAD1/4 A, SMAD1/4B, SMAD5/4 A, SMAD5/4B, SMAD5/4 C, SMAD8/4B, and SMAD8/4D (Fig. 5A). Interestingly, AMHY could also promote the activity of the *cyp19a1a* promoter by SMAD5/4D and SMAD8/4 A. The effect of AMH on the *cyp19a1a* promoter was also investigated, revealing that AMH could significantly inhibit the activity of the *cyp19a1a* promoter via a few combinations including SMAD1/4 A, SMAD1/4 C, and SMAD8/4 A, but promote the activity of *cyp19a1a* promoter by SMAD1/4B, SMAD1/4D, SMAD5/4 A, SMAD5/4D, SMAD8/4B, and SMAD8/4D (Fig. 5B). Overall, despite large differences between AMH and AMHY, AMHY maintained the enzymatic activity of AMH that can activate the Amhr2/SMADs signaling pathway. Moreover, AMHY has apparently evolved multiple new mechanisms to regulate the *cyp19a1a* signaling pathway. Thus, *amhy* can act as an upstream regulator of male sex determination.

### Different sex chromosome identified in Sinipercinae by GWAS

Previous studies have demonstrated that an XY sex determination system was present in *S. chuatsi*, *S. scherzeri,* and *S. knerii* (Yang et al. 2022). To further characterize the SDRs of *S. scherzeri*, genome re-sequencing analysis of ten male and nine female individuals was conducted. GWAS revealed that the sex-related SNPs aligned to the chromosome-level assembly of *S. chuatsi*. Interestingly, the sex related SNPs of *S. scherzeri* were also primarily located in the initial region of the *S. chuatsi* sex chromosome (Supplemental Fig. S14). Manhattan plot analysis of sex chromosomes revealed that significant sex related SNPs were primarily located in the initial region comprising about 2.5 Mbp (SDR), similar to that observed in *S. chuatsi.* More interestingly, a Y-specific *amh* sequence in the SDR was also identified and was located near the region with the most significant sex-related SNPs based on *Fst* analysis (Supplemental Fig. S15). Thus, the SDRs and SDGs of *S. chuatsi* and *S. scherzeri* were very similar. However, GWAS revealed sex related SNPs of *C. whiteheadi* that were primarily located on chromosome 11 of *S. chuatsi* (Supplemental Fig. S16 and S17). PCR analysis of *C. whiteheadi* sex-specific sequences demonstrated that an XY sex determination system was present in *C. whiteheadi*, with the sex chromosome being chromosome 11 (Supplemental Fig. S18). In addition, the SDR region did not harbor any Y-specific genes, with only one gene located 3 kbp upstream that was annotated as a phytanoyl-CoA hydroxylase-interacting protein-like gene (*Phyhipl*). These results revealed that evolution of the sex chromosome might have occurred in the ancestor of the genus *Siniperca*.

### Emergence of *amhy* predates species differentiation in genus *Siniperca*

**Fig. 6:**
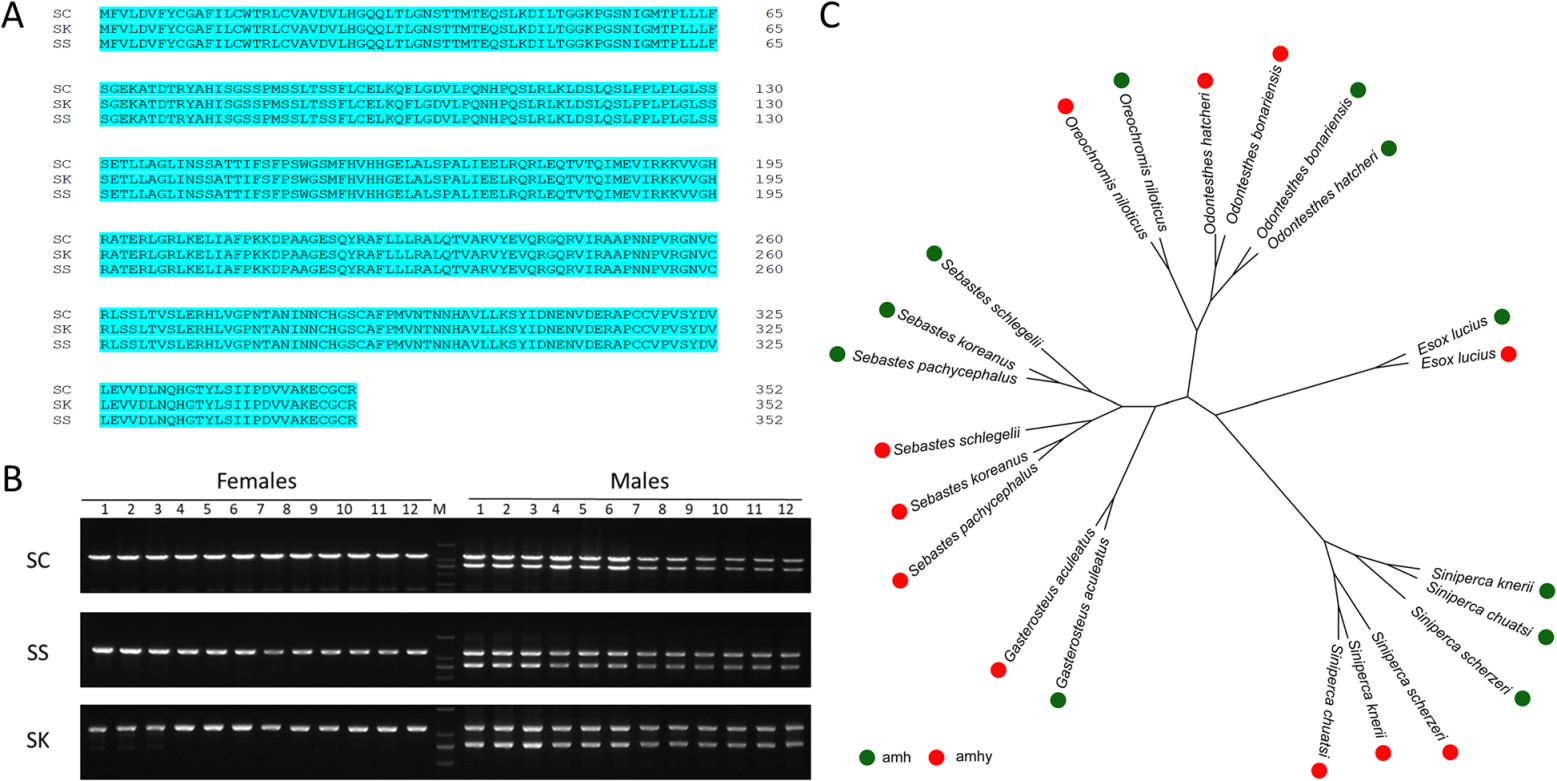
Evolutionary conservation of *amhy*. ***A*** The alignment of CDSs of *amhy* in *Siniperca chuatsi* (SC), *Siniperca scherzeri* (SS) and *Siniperca knerii* (SK). ***B*** PCR detection and genetic sexing using primers that span the exons of *amhy* in eight females and eight males of SC, SS and SK. ***C*** Phylogenetic analysis of *amh* and *amhy* from different fish species

A candidate *amhy* sequence of *S. scherzeri* was further identified based on the *amhy* sequence of *S. chuatsi*. Comparison by NCBI blast revealed a candidate *amhy* sequence was in the genome of male *S. knerii* (GCA_011952075.1). Alignment also revealed that the predicted *amhy* protein sequences of the three species were completely conserved (Fig. 6A). Even in the region of intron and promoter region, there were only a few differences (Supplemental Fig. S19 and S20). PCR validation confirmed that *amhy* was only present in male individuals of the three *Siniperca* species (Fig. 6B). Phylogenetic analysis further supported that *amhy* was derived from the replication of the *amh* gene of mandarin fish and other fish species (Fig. 6C). Timetree analysis subsequently revealed that *C. whiteheadi* diverged from the genus *Siniperca* about ~ 44–63 million years ago (Supplemental Fig. S21), consistent with the estimated divergence time of *amh* and *amhy* (~ 44 and ~ 51 million years ago). However, the high similarity of *amhy* in the three species suggests that *amhy* originated earlier than the speciation of the *Siniperca* genus and underwent strong purification selection. Thus, after gene translocation, *amhy* underwent rapid evolution due to recombination limitation before neospeciation in the genus *Siniperca*, but no change occurred after species differentiation.

### Duplication and transclocation of *amh* drives the origin and turnover of sex chromosomes in Sinipercinae

**Fig. 7:**
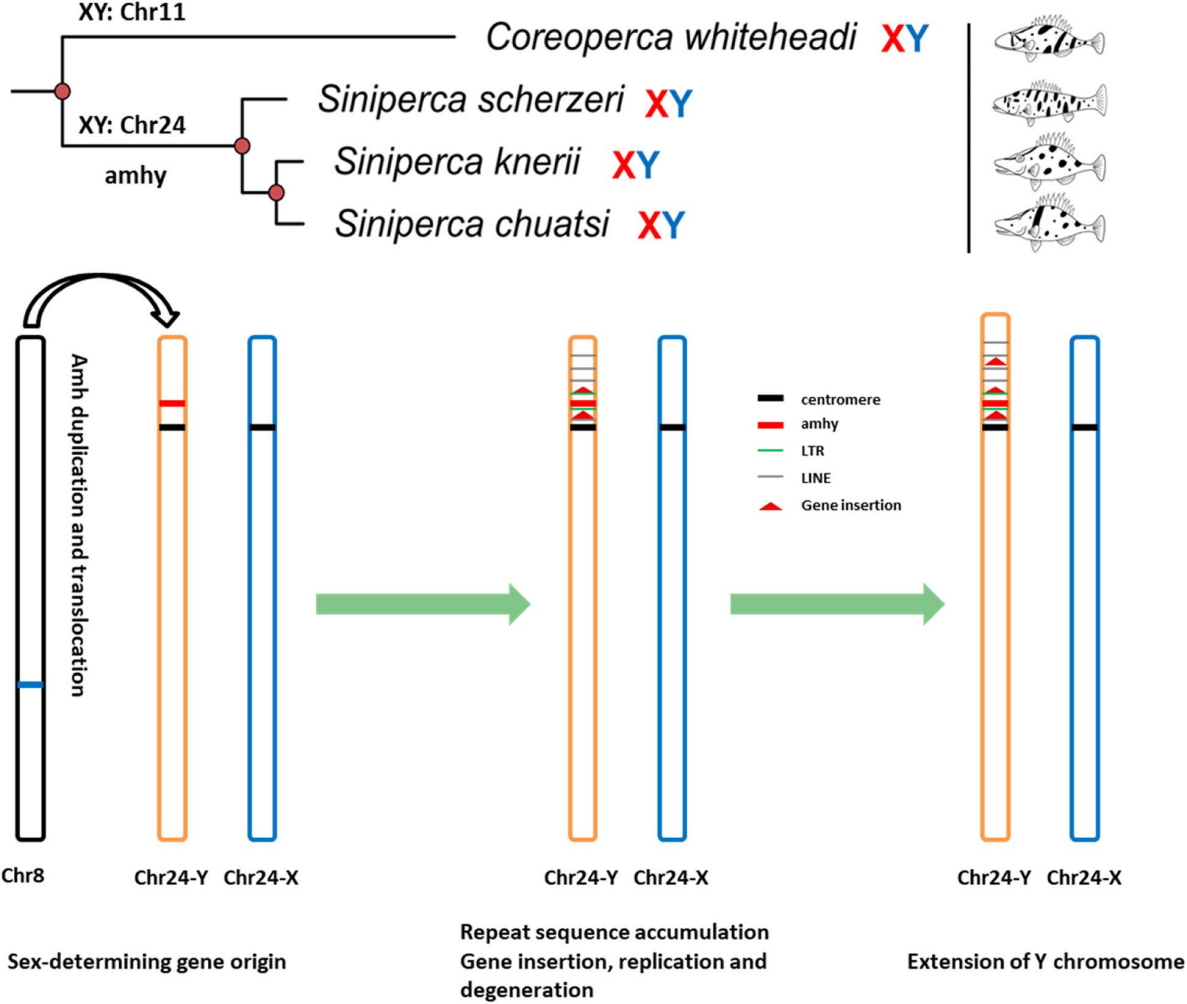
Schematic showing the origin and turnover of sex chromosome in Sinipercidae fishes

One additional sequence that was homologous to *amh* (*amhx*) was also identified in chromosome 20 (Supplemental Fig. S22). However, only the last exon of *amh* was highly similar to the *amhx* sequence, suggesting that *amh* may have migrated to this location, but then pseudogenized. Colinear analysis based on whole genomes of representative Perciformes species (including *S. chuatsi*) revealed that most chromosomes of these species exhibited highly conserved synteny, without obvious chromosome fusion except for *Micropterus salmoides* and *Gasterosteus aculeatus*. Surprisingly, *amh* duplication was also observed in *G*. *aculeatus*, *S. umbrosus,* and *C. lumpus*, although the chromosome distribution of *amh* duplication in these species was not identical, further suggesting frequent replication and translocation of *amh* in several Perciformes species (Supplemental Fig. S23 A). Combined with our previous research (Liu et al. 2023a, b), we found the adjacent genes to *amh* in the autosome did not transpose to the Y chromosome. In addition, slightly more *nlrc3* genes were enriched around *amhy* compared to the homologous region of the X chromosome (Supplemental Table S4) and LINEs were also significantly enriched in the adjacent region of SDGs compared to the corresponding regions of homologous genes in autosomes among three species (*S. chuatsi*, *G. aculeatus* (Peichel et al. 2020) and *Perca flavescens* (Feron et al. 2020)) with known sex chromosomes and SDGs (Supplemental Fig. S23B). Thus, *amhy* appears to have first duplicated and translocated to a proto-Y chromosome. Then, *amhy* was subjected to strong selection pressure leading to significant differentiation. Concomitantly, recombination inhibition spread to both sides of *amhy*, further causing accumulation of repetitive elements. This especially included LINEs alongside gene insertion, replication, and degeneration of the Y chromosome that gradually evolved into the extant extended Y chromosome (Fig. 7).

## Discussion

High-quality chromosome-level genomes are critical for understanding the origin and evolution of sex chromosomes. Fish are the most diverse group of vertebrates and are models for investigating the origin and evolution of vertebrates. A high-quality chromosome-level genome of mandarin fish was generated here, that when analyzed alongside genome resequencing data of *S. scherzeri* and *C. whiteheadi*, provided evidence that the male-specific supergene *amhy* duplicated from the autosomal *amh* that was located near a centromere. This duplication led to the combined limitation and accumulation of genes and repetitive elements, ultimately driving the formation of the extended Y chromosome in extant mandarin fish. Moreover, *amhy* appears to be a supergene that encodes completely identical proteins in other *Siniperca* species, consequently driving the origin and turnover of sex chromosomes in Sinipercidae. These results provide new support for the canonical model of sex chromosome evolution in vertebrates.

Sex chromosomes are highly heteromorphic and degenerate in mammals, but homomorphic and similar across most fish species. In zig-zag eel and yellow catfish, differences in gene compositions were not observed between X and Y chromosomes (Xue et al. 2021; Gong et al. 2023). In mandarin fish, the Y chromosome is at a relatively initial stage of degeneration, with retention of about 95.78% (522/545) of the genes present on the X chromosome. In addition, the SDR of mandarin fish is larger than in yellow catfish (0.3 Mbp) (Gong et al. 2023), but smaller than in zig-zag eel (7 Mbp) (Xue et al. 2021) and threespine stickleback fish (13.28 Mbp) (Peichel et al. 2020). In the threespine stickleback Y chromosome, only 44.1% of genes present on the X chromosome have been retained. Thus, it seems that no obvious correlation exists between the gene composition and SDR of the sex chromosome. Repetitive elements have been shown to play a central role in sex chromosome differentiation by causing insertion and duplication (Bachtrog et al. 2008; Ding et al. 2021), and are shown here to have also accumulated in the SDRs of these fish species. Highly similar genes and repetitive elements have been observed in the SDRs of X and Y chromosomes of mandarin fish, indicating limited recombination suppression in the past. Interestingly, among repetitive elements, LINEs are significantly enriched in the sex chromosomes and adjacent regions of SDGs. This phenomenon was also observed in mammalian X and avian Z chromosomes (Bailey et al. 2000; Melamed and Arnold 2009). Recently, LINE1 elements were shown to act as enhancers that regulate naïve pluripotency in embryonic stem cells and regulate genome compartmental organization (Meng et al. 2023), but also activate long-range gene expression (Li et al. 2024). Thus, the enrichment of LINE1 elements in the SDR may act as a regulator of sex related gene expression. This enrichment may also be the main driver of recombination suppression extension that further facilitates inversions and translocations (Wright et al. 2016). Notably, multiple *nlrc3* genes were found in the SDRs. NLRC3 proteins primarily participate in immune processes (Karki et al. 2016). Previous studies have suggested that duplicated genes on the sex chromosomes may be important for male fertility (Gvozdev et al. 2005) or provide a repair template through gene conversion to prevent degradation (Chang et al. 2018; Skinner et al. 2015). Thus, multiple *nlrc3* duplications may provide a repair template that has yet to degenerate and pseudogenize. Moreover, one *amhy* gene located near a centromere was successfully identified in the SDR. Like the *amhy* in northern pike (Pan et al. 2019), the *amhy* of *S. chuatsi* is also located near a centromere, supporting previous results that the vicinity of the centromere facilitates SDR formation (Xue et al. 2021; Zhang et al. 2008). According to the canonical model (Vicoso 2019), it is consequently reasonable to infer that the genomic segment containing the *amh* copy in *S. chuatsi* is duplicated and translocated into the adjacent region of the centromere of another autosome to form proto-Y chromosomes. The newly translocated genomic segment containing the *amh* copy can then halt recombination with the X chromosome due to a complete lack of homology. Subsequently, recombination suppression further causes gene replication and the accumulation of repetitive elements and pseudogenes. These processes are accompanied by the enlargement of the Y chromosome. Thus, the SDR would gradually enlarge and consequently form the sex chromosomes observed in extant fish.

Y-specific duplication of *amh* has been recently identified as a mechanism of sex determination based on gene knockout experiments in many fish species, including Patagonian pejerrey (*Odontesthes hatcheri*) (Hattori et al. 2012), Nile tilapia (*Orecochromis niloticus*) (Li et al. 2015), northern pike (*Esox lucius*) (Pan et al. 2019) and Japanese flounder (*Paralichthys olivaceus*) (Hattori et al. 2022), among others. Likewise, gene knockout and overexpression investigations in this study also demonstrated that *amhy* is a SDG in mandarin fish, suggesting *amhy* to be a conserved SDG in fish. Notably, *amhy* has also been identified as a candidate SDG in egg-laying mammals (monotremes) (Shearwin-Whyatt et al. 2024). Despite that *amhy* acts as a SDG in many species, the protein sequences of *amhy* vary considerably among species, which could be due to different selection pressures and/or divergence over time. Interestingly, regardless of variability, all *amhy* have retained the AMH and TGFβ domains. In fact, *amhy* is a truncated form of *amh* in mandarin fish, with loss of exons 2–4, suggesting that recombination limitation may have started at exon3, then spread laterally, while the important AMH domain remained in place. Despite that *amhy* is found in many fish species, such large fragment changes have not previously been reported. Even the TGFβ domains have highly variable amino acid residues when comparing *amh* and *amhy*, indicating potentially different affinities for their downstream receptors. In pufferfish, a single amino acid difference of *amhrII* in males leads to enhanced effects on the *amh* signaling pathway (Duan et al. 2021). Only one other study of tilapia *amh* and *amhy* has demonstrated inhibition of the *cyp19a1a* promotor activity by SMAD1/4B, which is due to only one variable amino acid (Liu et al. 2021). In this study, AMHY could inhibit the activity of the *cyp19a1a* promoter by multiple different combinations of SMADs from AMH, indicating a divergence of function between AMH and AMHY in mandarin fish. In addition, previous studies have shown that AMH alone could not modulate the mRNA expression of *cyp19a1a* (Sacchi et al. 2016; Liu et al. 2021). Nevertheless, the results of this study demonstrated that AMHY could directly inhibit the activity of the *cyp19a1a* promoter without the help of gonadotropin or other transcription factors like FOXL2 and SF1. More interestingly, the three *Siniperca* species analyzed here diverged approximately 8.06–11.56 million years ago, yet *amhy* has not differentiated at all in these species, which may suggest that the current form of *amhy* is highly effective in male sex determination. Further in-depth studies are needed to clarify functional differences between *amh* and *amhy*. Overall, the current study suggests that *amhy* is a highly potent male SDG that has been preserved in *Siniperca* fish.

Sex chromosome turnover frequently occurs in vertebrates. Fish are lower vertebrates, have highly homogeneous sex chromosomes, and are more prone to sex chromosome turnover compared to mammals and birds (Lichilín et al. 2021). Indeed, sex chromosome turnover in fish is usually caused by the creation of a new SDG or transposition of a sex-determining locus to an autosome (Meisel 2020). There are even different sex determination systems among different species within the same genus (Takehana et al. 2014; Einfeldt et al. 2021). Increasing numbers of fish sex chromosomes have been identified and assembled genomically, with many being relatively evolutionarily young compared to those of mammals and birds (El Taher et al. 2021; Pan et al. 2019). Thus, fish sex determination systems may not be stable and are prone to change. Among the Sinipercinae, the sex chromosome of *C. whiteheadi* was chromosome 11, with a small SDR that did not contain a SDG. In most fish species with known sex determination systems, no obvious SDGs have been identified (Li et al. 2022), which may suggest unstable mechanisms of sex determination. In the genus *Siniperca*, the sex chromosome has evolved into chromosome 24, while the SDG also evolved into *amhy*, indicating that sex chromosome turnover has happened in the ancestor of *Siniperca*. The dominance of sex chromosomes in tilapia has been established as Y > W > Z > X because WY, ZX, and XY individuals are males, while ZW individuals are females (Chen et al. 2018; Hulata et al. 1983), indicating that the Y chromosome that harbors *amhy* is a potent determinant of male sex. *Amhy* is by far the most common class of SDG found in fish (Pan et al. 2019). Further, *amhy* was identified as a candidate SDG in egg-laying mammals (monotremes), as discussed above (Shearwin-Whyatt et al. 2024). Thus, *amhy* appears to be a highly important SDG that has been retained in vertebrates. The combined results of this study suggest that sex chromosome turnover in Sinipercinae is primarily caused by replication and transposition of *amh* to chromosome 24. This process may represent a transition from an unstable to a stable Sex determination system. In all, these results demonstrate a classic evolutionary event consistent with the canonical model. Besides, the current form of *amhy* that is commonly shared by different species of genus *Siniperca* and more efficient inhibition of *cyp19a1a* gene expression than *amh*, is considered a supergene that has driven the origin and turnover of sex chromosomes in Sinipercidae fishes.

## Methods

### Ethics statement

Experiments were conducted in accordance with the guidelines and approval of the respective Animal Research and Ethics Committees of the Sun Yat-Sen University (SYSU-IACUC-2023-B1231).

### Whole genome sequencing and genome assembly

Genomic DNA was extracted from the muscle of an XY male *S. chuatsi* fish (Han et al. 2020). A long-read library was then constructed for Pacbio SMART sequencing according to the SMART bell Template Prep Kits. On-board sequencing was performed using the Pacbio Sequel II platform (Pacific Biosciences, USA) with the 20 kbp long-read layout. Illumina short-read sequencing data was generated on the Illumina Hiseq X-ten platform, as previously described (Han et al. 2020), and used for data correction. Fresh muscle tissue was also used for Hi-C library construction using the NEBNext Ultra II DNA library Prep Kit (NEB, USA) and by following the manufacturer instructions. The Hi-C library was also sequenced on the Illumina Hiseq X-ten platform.

The software program Canu (version 2.1.1; https://github.com/marbl/canu) was used for genome assembly (Koren et al. 2017). The genome size is set based on the genome evaluation results using Genome Scope 2.0 (Genome Size = 900 Mb). The minimum sequence length is set to be 1000 bp, and the minimum overlap length between sequences is set to be 500 bp. All other parameters are set to their default values.

The BWA aligner was used to align the Illumina short-read data to the assembled genome with default parameters and Pilon was used for three consecutive iterations of correction (Walker et al. 2014). Hi-C sequences were assembled using the Juicer assembly, combined with 3D-DNA analysis strategy (Yi and Charlesworth 2000). The chromosome sequences were further assembled according to the position relationships of the super scaffolds obtained by Hi-C sequencing. The integrity of the final assembly and the other two published assembly versions were assessed using the BUSCO software program (version 5.2.2) at the genome and chromosome levels, and with reference against the database Actinopterygii_odb10.

### Genome annotation

RNAs from multiple tissues (including brain, testis, ovaries, gills, livers, muscles, spleens, and kidneys) of *S. chuatsi* that met quality thresholds were mixed in equimolar amounts and subjected to Iso-seq analysis. The RNAs from early gonads at 5, 7, 10, 15, 20, 25, and 30 dah were also used for RNA-seq analysis on the Illumina NovaSeq6000 platform. The genome was further annotated using the PASA version 2.5.2 genome annotation pipeline, with the gmap and minimap2 alignment methods, max intron length of 100 kbp, in conjunction with run alternative splicing analysis and ab initio gene finding (Haas et al. 2008). The annotated genes were further used to search against the non-redundant protein sequences (Nr), the Uniprot protein, Kyoto Encyclopedia of Genes and Genomes (KEGG), Gene Ontology (GO), and Clusters of Orthologous Groups (COG) databases.

The RepeatModeler (version 2.0.3) software program was used to build a repeat model with default parameters. The RepeatMasker (version 4.1.2-p1) program was then used to identify repeat elements using the rmblastn search engine (http://www.repeatmasker.org). The distribution of repeat sequences was enumerated using an in-house python program, with 50 Kbp used as the statistical window and 25 kbp steps for statistical analyses and visualization. The identification of centromeres was performed according to previous analysis of the zig-zag eel and with reference to Cen-203 (Xue et al. 2021).

### Transcriptome analysis of early gonad

Gonad transcriptome analysis was also performed to characterize genes involved in early gonad development. Gene expression levels were calculated using Cuffdiff and Cuffnorm as previously described (Zhu et al. 2022). Gene differential expression analysis and correlation calculations were conducted as previously described (Liu et al. 2023a, b). Some gender-related differentially expressed genes of *S. chuatsi* were also identified based on previous analyses of sex-related genes (Li and Gui 2018; Tao et al. 2013). An expression heat map was used to visualize expressional differences. RT qPCR was also used to quantify *amhy* and well-known sex related genes like *amh*, *gsdf*, *dmrt1*, *foxl2,* and *cyp19a1a* in early gonad tissues. Moreover, gonad and tissue distribution analysis of *amh* and *amhy* were also conducted. The RT qPCR reactions and data analysis were performed as previously described (Han et al. 2021). All data are expressed as mean ± SD. Significant differences among groups were tested using t-test. *p* < 0.05 (*) indicates a significant difference. *p* < 0.01 (**) indicates a highly significant difference. *p* < 0.001 (***) indicates an extremely significant difference.

### Knockout of *amhy*

Genome editing technology via the CRISPR/Cas9 system was used to achieve gene knockout in mandarin fish. Based on the *amhy* transcript and genome sequence, CRISPR/Cas9 target sequences were identified using the ZiFiT platform (http://zifit.partners.org/ZiFiT/). gRNA preparation was performed as described previously (Li et al. 2014). EnGen® Spy Cas9 NLS (NEB, US) nuclease and *amhy*-gRNA were mixed and a small amount of phenol red without RNA enzyme was added to adjust the final concentrations of Cas9 nuclease and *amhy*-gRNA to 20 pmol/µL and 100 ng/µL, respectively. For mutation detection, PCR primers were designed according to the upstream and downstream sequences of the targeted editing site of *amhy*. PCR products were detected with T7 Endonuclease I (Vazyme, China) and Sanger sequencing method. Mature F0 individuals at 1 year old with a disrupted *amhy* sequence were used for in vitro fertilization with wild-type eggs. F1 individuals representing frame shift mutants of *amhy* were euthanized and dissected at 30 (*n* = 10) and 60 (*n* = 6) dah for histology and expression analysis. All data are expressed as mean ± SD. Significant differences among groups were tested using one-way analysis of variance, followed by Duncan's multiple tests, with SPSS version 21.0. *p* < 0.05 (*) indicates a significant difference. *p* < 0.01 (**) indicates a highly significant difference. *p* < 0.001 (***) indicates an extremely significant difference.

### Data access

The whole sequencing data generated in this study are available at the NCBI BioProject database (https://www.ncbi.nlm.nih.gov/bioproject/) under accession number PRJNA934159, PRJNA904906, PRJNA904515, PRJNA905482, PRJNA612372, PRJNA904821, PRJNA905868 and PRJNA905867.

## Supplementary Information

Supplementary Material 1.

Supplementary Material 2.

Supplementary Material 3.

Supplementary Material 4.

Supplementary Material 5.
