# Supplementary material for: Assembly and analysis of Sinipercidae fish sex chromosomes reveals that a supergene drives sex chromosome origin and turnover
Source: Adv Biotechnol (Singap). 2025 May 28;3(2):17. doi: 10.1007/s44307-025-00068-6 (PMC12119445; doi:10.1007/s44307-025-00068-6)
Supplement: Supplementary file 1 — Supplementary Material 1. [file 44307_2025_68_MOESM1_ESM.docx]

**Supplemental Methods**

**Fish sources and sample collection**

Mandarin fish used in this study were reared at the Guangdong Liangshi Aquatic Seed Industry Co., Ltd facility in Foshan City, Guangdong Province. *S. scherzeri*, *S. kneri*, and *C. whiteheadi* were obtained from Zhangjiajie, Hunan Province. The physical genders of fish were determined by visual assessment of their gonads. Fin or muscle tissue was excised and stored in liquid nitrogen or 95% ethanol. The tissues were then immediately stored in liquid nitrogen. In addition, gonads of male and female mandarin fish were separately collected after hatching at 5 (n=400), 7 (n=300), 10 (n=200), 13 (n=200), 15 (n=200), 18 (n=200), 20 (n=100), 25 (n=50), 30 (n=10), 60 (n=6), 90 (n=6), 120 (n=6), 150 (n=6), 180 (n=6) and 360 (n=6) dah, and stored in RNA Keeper Tissue Stabilizer (Vazyme, China) and Bouin’s solution. Fish were anaesthetized using MS222 to minimize suffering.

**Identification of sex chromosome-specific sequences**

Y chromosome-specific molecular markers of *S. chuatsi* that we previously identified were selected for nucleic acid sequence alignment to identify possible Y chromosome characteristic sequences (Han et al. 2020). Moreover, X chromosome characteristic sequences were also identified based on sequencing depth and homology with Y chromosomes for auxiliary screening (Han et al. 2021). X chromosome and Y chromosome sequence fragments were obtained using 10 kbp windows and 5 kbp steps. Then, BLASTn nucleic acid sequence alignment was used to align X chromosome sequence fragments to the Y chromosome (E-value = 1E-50, identity > 80%, sequence length > 1000 bp), while conversely comparing the Y chromosome sequence fragments to the X chromosome in the same manner.

**SDR characterization**

Illumina sequencing data for eight female and eight male *S. chuatsi* was subjected to sequence alignment to the genome using the BWA program (version 0.7.17-R1188) with double-end alignment, a base error rate set to 0.04, and other parameters set to defaults. Sequence data were aligned to the assembled chromosome-level genome of *S. chuatsi* and the SAMtools program (version 1.9) was used to sort aligned sequences. The Deepvariant software program (release 1.3.0) was used to detect population SNPs. The SAMtools, Mpileup and Python programs were used to review SNP genotypes, base sequencing quality, read alignment quality, and comprehensively determine SNP polymorphisms. Correlation analysis was conducted between SNP datasets and gender, while *p*-value calculations were conducted using the TASSEL5 (version: 5.2.80) analysis software, after removing SNP loci with minor allele frequency (MAF) values < 0.05. A general linear model (GLM) was used to analyze relationships between male and female populations. Visualization was conducted using -log10 (p), a window of 50 kbp, a step of 25 kbp, and a window with the number of SNPs greater than or equal to 10 was selected for visualization. The distribution map within the chromosome was visualized without screening the number of SNPs in the window. The *Fst* (fixation coefficient) values were calculated using the hierfstat package for R (version: 0.04–22) with basic.stats methods (ploidy = 2). During the calculation, the samples were divided into two groups: females and males. The weighted *Fst* value statistic window was 50 kbp and the step was 25 kbp. The calculation of nucleic acid distance was based on BLAST (version: 2.15.0+) alignment. The X chromosome sequence was analyzed over windows of 20 Kbp and sequence fragments were obtained by 10 Kbp in each step. BLASTn nucleic acid alignment was used to align the X fragment sequences to the Y chromosome. If the X sequence did not match the Y sequence at all, the nucleic acid distance was defined as 1. After comparison, the proportion of the number of unmatched bases between sequences (including: mismatches, gaps, and the area of the comparison) over the whole window was calculated, the value of the nucleotide distance was calculated, and the distribution map of nucleic acid distances was visualized.

Illumina sequencing data for ten male and nine female *S. scherzeri,* in addition to ten male and ten female *C. whiteheadi* were used for GWAS to identify their sex chromosomes and characterize their SDRs. A primer pair that spans the first exon and the second exon of *amhy* was designed based on the CDS and genomic sequences of *amhy* in the three *Siniperca* species. Further, a primer pair was designed based on identified sex-specific sequences of *C. whiteheadi* and used for PCR amplification in female and male *C. whiteheadi*. PCR products were subsequently visualized with agarose electrophoresis.

**Cloning and sequence analysis**

To clone *amhy*, a HiScript III 1St Strand cDNA Synthesis Kit (Norvezan, China) was used to reverse transcribe the testis RNA template from male fish. The open reading frame (ORF) of *amhy* was amplified with primers (Supplementary Supplemental Table S11) that were designed according to the genome and transcriptome sequences. To obtain the complete transcripts of the *amh* and *amhy* genes, RACE (Rapid Amplification of cDNA ends) primers were also designed based on the obtained *amh* and *amhy* transcript sequences and used for 5 'and 3' RACE PCR. The testis RNA template of male fish exhibited high RNA integrity and was used to prepare the RACE template using the SMARTer RACE cDNA Amplification Kit (Clontech, USA). The domain composition, sequence alignments, and expression analysis were all performed as previously described (Han et al. 2021).

Structural prediction of the intracellular domains of *amh* and *amhy* was performed using the SWISS-MODEL. *Acanthopagrus schlegelii* (Black porgy) AMH (PDB accession number: D2XUR9.1.A) yielded the best model. The resulting 3D models were visualized with the PyMOL Molecular Graphics System (v2.7; Schrodinger, LLC), and the amino acid sites of the TGFβ domain that differed between AMH and AMHY were identified. The protein interaction sites between AMH and AMHRII, AMHY and AMHRII were predicted using Alpha Fold 3. However, AMHRII is a transmembrane protein. Thus, the interaction sites located in extracellular region (131 amino acids in N terminal of AMHRII) and the interaction sites distributed in TGFβ region of AMH and AMHY were more reliable. Besides, the predicted transcription factor binding sites locates on *amhy* promoter and *amh* promoter (about 3000 bp upstream ATG) were predicted and compared using ALGGEN.

**Overexpression of amhy**

In addition, the CDS of *amhy* was cloned into the pcDNA4.0 vectors (Invitrogen, Carlsbad, CA). The plasmids were then encapsulated by liposomes. The preparation of liposomes was conducted as previously described, with minor modifications (Han et al. 2018). The all-female mandarin fish were randomly divided into two groups at 15 dah: those fed with the empty plasmid (pcDNA4.0) (n = 30), and those fed the *amhy* overexpression plasmid (pcDNA4.0-*amhy*) (n = 30). The individuals were cultured in water tanks and fed with bait fish twice a day. The bait fish were first fed a diet containing an empty plasmid (1 ml/kg feed) or the *amhy* overexpression plasmid (1 ml/kg feed). The bait fish were then immediately fed to the mandarin fish (Liu et al. 2021).

**Phylogenetic analysis and molecular clock analysis**

Single copy orthologous genes were identified from *S. chuatsi*, *Gasterosteus aculeatus*, *Micropterus dolomieu*, *Micropterus salmoides*, and *Scortum barcoo* using the OrthoFinder software program (version 2.5.4). The dendroblast method was used for gene tree inference using the diamond sequence alignment program, and with other default parameters (Emms and Kelly 2019). A total of 2,875 single copy orthologous gene sequences were identified across 36 species. Coding gene sequence alignment was conducted using the MAFFT sequence alignment software program (version v7.490). The comparison parameter was set to the maximum number of iterative refinement levels (1,000) and the comparison was performed using the localpair method. The gappyout method of trimAl (version v1.4 rev15) was used to filter the gaps of sequence alignment. IQ-TREE (version 2.0.5) was then used to conduct substitution model selection and phylogenetic reconstruction (Minh et al. 2020). The optimal substitution model for each gene was selected using the IQ-TREE ModelFinder algorithm and the model with the smallest BIC value was selected as the optimal model. The species tree was constructed with tree topology assessed with 1,000 ultrafast bootstraps, and 1,000 replicates for the SH approximate likelihood ratio test. Figtree (v1.4.4) was used for visualization of the tree. Molecular clock split time analysis was conducted using BEAST2 v2.7.1, using the HKY+G/GTR+G substitution model. Given the lack of adequate fossils, phylogenetic calibration times were estimated using Timetree with the divergence of (*Macquaria ambigua*-*Macquaria australasica*) set as 22.38 MYA. A total of 10,000,000 iterations were used for the dating analysis. The lineage-specific median Ks value was estimated between *S. chuatsi* and *Micropterus salmoides* based on full-length cDNA sequences. The Ks value for the comparison of *amh* and *amhy* of *S. chuatsi* was calculated using the KaKs_Calculator 2.0 (Wang et al. 2010) following the NG method and by using a standard model. The approximate divergence time between *amh* and *amhy* was estimated assuming that *amh* and *amhy* independently evolved at a rate equal to the lineage-specific rate of divergence (Chen et al. 2014).

**Transfection experiments**

The complete CDSs of *amh, amhrII,* and *smad1/5/8/4a/4b/4c/4d* were cloned into the pcDNA4.0 (+) expression vector. In addition, the promotor sequences of cyp19a1a were subcloned into the pGL4.1 vector. The promoter sequences originate from about 3.0 kbp upstream of the start codon ATG. HEK 293T cells were cultured in 48-well culture plates in DMEM (High Glucose) (Gibco, USA) with 10% fetal bovine serum at 37°C and with a 5% CO_2_ atmosphere. Cell transfection was performed using the ViaFect™ transfection reagent (Promega, USA).

To explore whether AMH/AMHY regulates the activity of the *Cyp19a1a* promoter through the AmhrII/Smads signaling pathway, transfection assays were conducted including 20 μl of Opti-MEM, 5 ng of the pRL-TK internal reference plasmid, 200 ng of cyp19a1a-pGL4.1, 50 ng of amhrII-pcDNA4.0, smad1/5/8-pcDNA4.0, and smad4a/4b/4c/4d-pcDNA4.0, all in a mixture with 0, 100, and 200 ng of amh/amhy-pcDNA4.0 supplemented with empty pcDNA4.0 plasmid at a 555 ng plasmid mass. Each reaction was added to a well of a plate and cultured for 18 h. Finally, luciferase activity was measured using a Dual Luciferase Reporter Assay Kit (Vazyme, China) according to the manufacturer’s instructions. All data are expressed as mean ± SD. Significant differences among groups were tested using one-way analysis of variance, followed by Duncan's multiple tests, with SPSS version 21.0. p < 0.05 (*) indicates a significant difference. p < 0.01 (**) indicates a highly significant difference. p < 0.001 (***) indicates an extremely significant difference.
