# Supplementary material for: Assembly and analysis of Sinipercidae fish sex chromosomes reveals that a supergene drives sex chromosome origin and turnover
Source: Adv Biotechnol (Singap). 2025 May 28;3(2):17. doi: 10.1007/s44307-025-00068-6 (PMC12119445; doi:10.1007/s44307-025-00068-6)
Supplement: Supplementary file 2 — Supplementary Material 2. [file 44307_2025_68_MOESM2_ESM.docx]

**Supplementary Table S1.** Pacbio long read, Illumina short read, and Hi-C sequencing data for *S*. *chuatsi*.

| Library type | Read number |  | Base(Gb)/depth(X) |  |
| --- | --- | --- | --- | --- |
|  | XX | XY | XX | XY |
| Long reads | 0 | 5,411,841 | 0 | 95.96/98.72 |
| Hi-C | 0 | 532,950,608 | 0 | 79.94/82.24 |
| Short reads | 389,388,480 | 356,370,370 | 58.41/60.03 | 53.46/55.00 |

**Supplementary Table S2.** Chromosome length statistics for mandarin fish.

| Chrom | A(bp) | T(bp) | C(bp) | G(bp) | N(bp) | GC(%) | Length(bp) | From contigs |
| --- | --- | --- | --- | --- | --- | --- | --- | --- |
| Chr1 | 11,545,978 | 11,543,535 | 7,774,469 | 7,754,512 | 84,401 | 40.21 | 38,702,895 | 79 |
| Chr2 | 11,068,274 | 11,065,788 | 7,503,099 | 7,520,118 | 110,051 | 40.43 | 37,267,330 | 72 |
| Chr3 | 11,697,614 | 11,695,959 | 8,053,683 | 8,044,055 | 47,225 | 40.76 | 39,538,536 | 72 |
| Chr4 | 10,591,540 | 10,592,273 | 7,191,614 | 7,137,676 | 79,498 | 40.35 | 35,592,601 | 65 |
| Chr5 | 10,308,003 | 10,326,154 | 6,981,677 | 6,983,524 | 39,978 | 40.36 | 34,639,336 | 51 |
| Chr6 | 9,984,594 | 10,044,654 | 6,825,180 | 6,817,022 | 62,028 | 40.52 | 33,733,478 | 57 |
| Chr7 | 10,030,458 | 9,996,222 | 6,817,952 | 6,795,338 | 41,302 | 40.47 | 33,681,272 | 64 |
| Chr8 | 10,081,148 | 10,041,197 | 6,920,953 | 6,967,197 | 73,677 | 40.84 | 34,084,172 | 53 |
| Chr9 | 9,667,938 | 9,702,045 | 6,549,490 | 6,577,052 | 41,464 | 40.39 | 32,537,989 | 47 |
| Chr10 | 9,669,023 | 9,684,617 | 6,568,243 | 6,575,202 | 49,570 | 40.44 | 32,546,655 | 30 |
| Chr11 | 9,480,417 | 9,478,569 | 6,451,385 | 6,447,291 | 27,396 | 40.49 | 31,885,058 | 52 |
| Chr12 | 9,201,588 | 9,165,610 | 6,208,808 | 6,212,659 | 37,227 | 40.35 | 30,825,892 | 60 |
| Chr13 | 9,348,949 | 9,338,689 | 6,347,078 | 6,324,371 | 45,295 | 40.41 | 31,404,382 | 43 |
| Chr14 | 8,865,936 | 8,897,341 | 5,921,198 | 5,916,592 | 21,611 | 39.99 | 29,622,678 | 33 |
| Chr15 | 9,001,552 | 8,971,976 | 6,186,450 | 6,184,700 | 56,855 | 40.77 | 30,401,533 | 47 |
| Chr16 | 8,913,544 | 8,926,547 | 6,096,608 | 6,124,995 | 37,184 | 40.65 | 30,098,878 | 50 |
| Chr17 | 8,769,526 | 8,761,797 | 5,972,669 | 5,954,172 | 33,525 | 40.49 | 29,491,689 | 50 |
| Chr18 | 8,740,744 | 8,756,703 | 5,965,557 | 5,967,911 | 32,585 | 40.55 | 29,463,500 | 38 |
| Chr19 | 8,387,875 | 8,399,052 | 5,605,450 | 5,602,162 | 35,094 | 40.03 | 28,029,633 | 35 |
| Chr20 | 8,565,095 | 8,582,272 | 5,903,097 | 5,884,008 | 38,078 | 40.74 | 28,972,550 | 28 |
| Chr21 | 8,079,290 | 8,083,158 | 5,574,331 | 5,552,552 | 38,244 | 40.78 | 27,327,575 | 45 |
| Chr22 | 7,441,215 | 7,370,276 | 5,045,602 | 5,042,843 | 56,367 | 40.51 | 24,956,303 | 49 |
| Chr23 | 7,387,483 | 7,335,273 | 5,053,259 | 5,057,437 | 32,511 | 40.72 | 24,865,963 | 48 |
| ChrX | 5,928,167 | 5,878,256 | 4,079,321 | 4,065,656 | 32,716 | 40.83 | 19,984,116 | 43(6) |
| ChrY | 6,059,296 | 6,026,105 | 4,159,006 | 4,179,749 | 31,816 | 40.82 | 20,455,972 | 42(5) |
| other | 18,742,641 | 18,719,674 | 13,847,927 | 13,813,843 | 0 | 42.48 | 65,124,085 | / |
| Haplotype genome of XX | 222,755,951 | 222,637,963 | 151,597,173 | 151,509,045 | 1,153,882 | 972 | 749,654,014 | / |
| Haplotype genome of XY | 222,887,080 | 222,785,812 | 151,676,858 | 151,623,138 | 1,152,982 | 972 | 750,125,870 | / |

**Supplementary Table S3.** Genome completeness assessments based on BUSCO analysis.

|  |  |  | Complete BUSCOs (C) | Complete and single-copy BUSCOs (S) | Complete and duplicated BUSCOs (D) | Fragmented BUSCOs (F) | Missing BUSCOs (M) | Total BUSCO groups searched |
| --- | --- | --- | --- | --- | --- | --- | --- | --- |
| This project | Genome | Number | 3592 | 3353 | 239 | 15 | 33 | 3640 |
|  |  | Ratio(%) | 98.68 | 92.12 | 6.57 | 0.41 | 0.91 |  |
|  | Chr1-23, X, Y | Number | 3583 | 3538 | 45 | 14 | 43 | 3640 |
|  |  | Ratio(%) | 98.43 | 97.20 | 1.24 | 0.38 | 1.18 |  |
| He et al. 2020 | Genome | Number | 3590 | 3549 | 41 | 19 | 31 | 3640 |
|  |  | Ratio(%) | 98.63 | 97.50 | 1.13 | 0.52 | 0.85 |  |
|  | LG1-24 | Number | 3582 | 3552 | 30 | 20 | 38 | 3640 |
|  |  | Ratio(%) | 98.41 | 97.58 | 0.82 | 0.55 | 1.04 |  |
| Yang et al. 2022 | Genome | Number | 3570 | 3549 | 21 | 12 | 58 | 3640 |
|  |  | Ratio(%) | 98.08 | 97.50 | 0.58 | 0.33 | 1.59 |  |
|  | LG1-24 | Number | 3570 | 3549 | 21 | 12 | 58 | 3640 |
|  |  | Ratio(%) | 98.08 | 97.50 | 0.58 | 0.33 | 1.59 |  |

**Supplementary Table S4**. Gene composition of X and Y special regions. Red letters indicate Y specific genes and blue letters indicate X specific genes.

**Supplementary Table S5.** Pseudogene distribution statistics for different chromosomes.

| Chromosome | Pseudogene | Length(bp) | Pseudogene per Mb |
| --- | --- | --- | --- |
| Chr1 | 45 | 38702895 | 1.16 |
| Chr2 | 89 | 37267330 | 2.39 |
| Chr3 | 117 | 39538536 | 2.96 |
| Chr4 | 50 | 35592601 | 1.40 |
| Chr5 | 53 | 34639336 | 1.53 |
| Chr6 | 60 | 33733478 | 1.78 |
| Chr7 | 40 | 33681272 | 1.19 |
| Chr8 | 68 | 34084172 | 2.00 |
| Chr9 | 52 | 32537989 | 1.60 |
| Chr10 | 49 | 32546655 | 1.51 |
| Chr11 | 44 | 31885058 | 1.38 |
| Chr12 | 59 | 30825892 | 1.91 |
| Chr13 | 60 | 31404382 | 1.91 |
| Chr14 | 28 | 29622678 | 0.95 |
| Chr15 | 90 | 30401533 | 2.96 |
| Chr16 | 48 | 30098878 | 1.59 |
| Chr17 | 79 | 29491689 | 2.68 |
| Chr18 | 62 | 29463500 | 2.10 |
| Chr19 | 40 | 28029633 | 1.43 |
| Chr20 | 83 | 28972550 | 2.86 |
| Chr21 | 39 | 27327575 | 1.43 |
| Chr22 | 29 | 24956303 | 1.16 |
| Chr23 | 77 | 24865963 | 3.10 |
| ChrX | 60 | 19984116 | 3.00 |
| X-link region | 24 | 2000000 | 12.00 |
| ChrY | 83 | 20455972 | 4.06 |
| Y-link region | 47 | 2380000 | 19.75 |
| ChrXY-common region | 36 | 17984116 | 2.00 |
| Genome | 1611 | 792474102 | 2.03 |

**Supplementary Table S6.** SNP distribution statistics for the whole genome.

| Chrom | GC(%) | Length(bp) | SNPs | SNPs/Kb |
| --- | --- | --- | --- | --- |
| Chr1 | 40.21 | 38,702,895 | 190,768 | 4.93 |
| Chr2 | 40.43 | 37,267,330 | 313,579 | 8.41 |
| Chr3 | 40.76 | 39,538,536 | 236,364 | 5.98 |
| Chr4 | 40.35 | 35,592,601 | 200,144 | 5.62 |
| Chr5 | 40.36 | 34,639,336 | 190,407 | 5.50 |
| Chr6 | 40.52 | 33,733,478 | 189,666 | 5.62 |
| Chr7 | 40.47 | 33,681,272 | 168,100 | 4.99 |
| Chr8 | 40.84 | 34,084,172 | 225,850 | 6.63 |
| Chr9 | 40.39 | 32,537,989 | 243,080 | 7.47 |
| Chr10 | 40.44 | 32,546,655 | 168,960 | 5.19 |
| Chr11 | 40.49 | 31,885,058 | 198,504 | 6.23 |
| Chr12 | 40.35 | 30,825,892 | 222,643 | 7.22 |
| Chr13 | 40.41 | 31,404,382 | 170,692 | 5.44 |
| Chr14 | 39.99 | 29,622,678 | 150,569 | 5.08 |
| Chr15 | 40.77 | 30,401,533 | 204,011 | 6.71 |
| Chr16 | 40.65 | 30,098,878 | 169,051 | 5.62 |
| Chr17 | 40.49 | 29,491,689 | 173,417 | 5.88 |
| Chr18 | 40.55 | 29,463,500 | 201,192 | 6.83 |
| Chr19 | 40.03 | 28,029,633 | 160,413 | 5.72 |
| Chr20 | 40.74 | 28,972,550 | 273,590 | 9.44 |
| Chr21 | 40.78 | 27,327,575 | 166,345 | 6.09 |
| Chr22 | 40.51 | 24,956,303 | 145,599 | 5.83 |
| Chr23 | 40.72 | 24,865,963 | 222,528 | 8.95 |
| ChrX | 40.83 | 19,984,116 | 175,200 | 8.77 |
| ChrY(2Mb) | 40.82 | 20,455,972 | 15,885 | 0.78 |
| other | 42.48 | 65,124,085 | / | / |
| Genome | 40.6 | 835,234,071 | 4,776,557 | 5.72 |

**Supplementary Table S7.** Results of genotyping sex detection in phenotypic male and female mandarin fish based on *amhy* sequences.

|  |  | genotype |  |
| --- | --- | --- | --- |
| Phenotype | Female | Male | Sum |
| Female | 120 | 0 | 120 |
| Male | 0 | 120 | 120 |
| Sum | 120 | 120 | 240 |

**Supplementary Table S8.** Repetitive element composition for each chromosome.

**Supplementary Table S9.** The predicted transcription factor binding sites locates on *amhy* promoter but not *amh* promoter.

| Factor name | Start position | End position | Dissimilarity | String | RE equally | RE query |
| --- | --- | --- | --- | --- | --- | --- |
| AhR:Arnt [T05394] | 1498 | 1507 | 11.132 | CCACGCATGG | 0.168 | 0.096 |
| AhR:Arnt [T05394] | 1666 | 1675 | 11.132 | CCACGCATGG | 0.168 | 0.096 |
| AhR:Arnt [T05394] | 3371 | 3380 | 9.115 | CCACGCAAGG | 0.340 | 0.149 |
| ATF-1 [T00968] | 2604 | 2615 | 9.791 | GGCAGGACGTCA | 0.017 | 0.011 |
| ATF-1 [T00968] | 3203 | 3214 | 9.373 | TGACGTAACTGG | 0.007 | 0.004 |
| BTEB4 [T05053] | 1129 | 1137 | 8.345 | GGGGCTGAA | 0.129 | 0.061 |
| BTEB4 [T05053] | 1162 | 1170 | 7.162 | GGGGCTGGA | 0.388 | 0.145 |
| BTEB4 [T05053] | 1373 | 1381 | 7.162 | TGCAGCCCC | 0.388 | 0.145 |
| BTEB4 [T05053] | 1950 | 1958 | 12.330 | GGGGCTATG | 0.311 | 0.105 |
| BTEB4 [T05053] | 2949 | 2957 | 13.327 | GGGGCAATA | 0.414 | 0.194 |
| BTEB4 [T05053] | 3014 | 3022 | 10.245 | GGGGCATGT | 0.311 | 0.141 |
| CACCC-binding factor [T00077] | 1467 | 1478 | 8.075 | GGCACCACCCCC | 0.037 | 0.019 |
| CACCC-binding factor [T00077] | 2058 | 2069 | 14.347 | TTGGGTGGTACT | 0.090 | 0.044 |
| core-binding factor [T01530] | 1731 | 1742 | 0.983 | GAAAACCACAGC | 0.001 | 0.001 |
| CREBbeta [T02361] | 2606 | 2615 | 13.361 | CAGGACGTCA | 0.680 | 0.579 |
| CREBbeta [T02361] | 2608 | 2617 | 13.561 | GGACGTCATT | 0.680 | 0.579 |
| CREBbeta [T02361] | 3201 | 3210 | 13.876 | ATTGACGTAA | 0.408 | 0.382 |
| CREBbeta [T02361] | 3203 | 3212 | 4.486 | TGACGTAACT | 0.026 | 0.033 |
| CRE-BP2 [T01017] | 2608 | 2615 | 13.586 | GGACGTCA | 1.087 | 0.961 |
| CRE-BP2 [T01017] | 3203 | 3210 | 13.586 | TGACGTAA | 1.087 | 0.961 |
| CREMalpha [T01803] | 3199 | 3210 | 9.513 | GGATTGACGTAA | 0.089 | 0.102 |
| FXR:RXR-alpha [T05318] | 992 | 1003 | 14.724 | ACCTAAGGGTAA | 0.237 | 0.235 |
| GAGA factor [T00301] | 1193 | 1200 | 4.032 | CGCTCAAC | 0.207 | 0.154 |
| GAGA factor [T00301] | 1527 | 1534 | 6.023 | CGCTCCAG | 0.414 | 0.214 |
| GAGA factor [T00301] | 3311 | 3318 | 3.474 | CGCTCATT | 0.621 | 0.302 |
| GAGA factor [T00301] | 684 | 691 | 3.474 | AATGAGCG | 0.621 | 0.302 |
| IA-1 [T05887] | 1374 | 1386 | 12.276 | GCAGCCCCTTGAC | 0.035 | 0.011 |
| MAZ [T00490] | 1452 | 1462 | 14.730 | CCCTCCATCCT | 0.238 | 0.072 |
| MAZ [T00490] | 1521 | 1531 | 1.657 | CCCTCCCGCTC | 0.028 | 0.009 |
| MAZ [T00490] | 1614 | 1624 | 14.392 | CCTTCCCCCTC | 0.238 | 0.072 |
| MAZ [T00490] | 1689 | 1699 | 1.860 | CCCTCCCATTC | 0.051 | 0.019 |
| MAZ [T00490] | 3170 | 3180 | 14.730 | CCCTCTCTCTC | 0.238 | 0.072 |
| MAZ [T00490] | 656 | 666 | 14.713 | CCTTCCCCCAC | 0.238 | 0.072 |
| Pax-2.1 [T03031] | 1482 | 1492 | 13.850 | TTCACCCAGGG | 0.131 | 0.055 |
| Pax-2.1 [T03031] | 3370 | 3380 | 14.808 | GCCACGCAAGG | 0.146 | 0.067 |
| Pax-2.2 [T03219] | 1430 | 1439 | 8.695 | TGATGCGTCC | 0.379 | 0.216 |
| Pax-9a [T03593] | 1430 | 1439 | 8.695 | TGATGCGTCC | 0.379 | 0.216 |
| Pax-9b [T03594] | 1430 | 1439 | 8.695 | TGATGCGTCC | 0.379 | 0.216 |
| PPAR-alpha:RXR-alpha [T05221] | 1146 | 1156 | 9.158 | TGGACCCAGAG | 0.087 | 0.029 |
| PPAR-alpha:RXR-alpha [T05221] | 1179 | 1189 | 5.286 | TCGACCCAGTG | 0.060 | 0.028 |
| Sp3 [T02338] | 1129 | 1137 | 6.939 | GGGGCTGAA | 0.259 | 0.098 |
| Sp3 [T02338] | 1162 | 1170 | 1.993 | GGGGCTGGA | 0.078 | 0.024 |
| Sp3 [T02338] | 1373 | 1381 | 7.645 | TGCAGCCCC | 0.104 | 0.037 |
| Sp3 [T02338] | 1950 | 1958 | 10.874 | GGGGCTATG | 0.401 | 0.164 |
| Sp3 [T02338] | 2949 | 2957 | 12.130 | GGGGCAATA | 0.337 | 0.165 |
| Sp3 [T02338] | 3014 | 3022 | 7.184 | GGGGCATGT | 0.259 | 0.098 |
| SRF (504 AA) [T00765] | 1050 | 1062 | 12.555 | GCATATTAGGAAG | 0.036 | 0.040 |
| SRF (504 AA) [T00765] | 877 | 889 | 12.555 | GCATATTAGGAAG | 0.036 | 0.040 |
| TFE3-S [T00814] | 2111 | 2122 | 5.660 | GGCATGTGGGGG | 0.033 | 0.015 |
| TFE3-S [T00814] | 676 | 687 | 12.452 | AACATGTGAATG | 0.109 | 0.124 |
| YY1 [T00278] | 3254 | 3264 | 3.215 | CTCGGCCATCC | 0.021 | 0.009 |

**Supplementary Table S10.** Sequence length and identity of *amh* and *amhy* in different fish species.

| Species | CDS length of *amh/amhy* | Number of amino acid of *amh/amhy* | Identity of CDS | Identity of amino acid sequence |
| --- | --- | --- | --- | --- |
| *Esox lucius* | 1674/1680 | 577/559 | 79.94% | 68.91% |
| *Odontesthes hatcheri* | 1545/1548 | 514/515 | 95.55% | 92.05% |
| *Odontesthes bonariensis* | 1533/1545 | 510/514 | 94.52% | 90.89% |
| *Sebastes schlegelii* | 1593/1593 | 530/530 | 95.42% | 92.08% |
| *Sebastes koreanus* | 1593/1593 | 530/530 | 94.98% | 91.13% |
| *Sebastes pachycephalus* | 1593/1593 | 530/530 | 95.23% | 91.89% |
| *Oreochromis niloticus* | 1545/1545 | 514/514 | 99.94% | 99.81% |
| *Gasterosteus aculeatus* | 1569/1581 | 522/526 | 77.55% | 66.16% |
| *Siniperca chuatsi* | 1059/1665 | 554/352 | 52.52% | 50.90% |

**Supplementary Table S11.** Estimation of divergence times for *amh* and *amhy*.

| KaKs statistics at the genome level | Genome | | | Time (MYA) | | Ks change/size/My | |
| --- | --- | --- | --- | --- | --- | --- | --- |
|  | Ka | | Ks | From | To | Min | Max |
| *Siniperca chuatsi* vs *Micropterus salmoides* | 0.038 | | 0.157 | 74.86 | 86.72 | 0.0018 | 0.0021 |
| Separation time of *amh* and *amhy* was calculated based on KaKs information | Gene | | | Time (MYA) | |  |  |
|  | Ka | Ks | | From | To | combined divergence rate (change/size/My) | |
| *Siniperca chuatsi* *amh* vs *amhy* | 0.104 | 0.186 | | 44.35 | 51.37 | 0.0036 | 0.0042 |

**Supplementary Table S12.** The predicted protein interaction sites between AMH and AMHRII, AMHY and AMHRII by alphafold 3.

| AMH and AMHRII | | | | AMHY and AMHRII | | | |
| --- | --- | --- | --- | --- | --- | --- | --- |
| AMHRII | | AMH | | AMHRII | | AMHY | |
| intracelluar section | extracelluar section | TGFβ domain | other region | intracelluar section | extracelluar section | TGFβ domain | other region |
| Q298 | Q35 | Y539 | R458 | S382 | Q35 | L338 | A249 |
| D332 | N36 | T538 | N455 | N384 | N36 | Y337 | R245 |
| N447 | Y39 | H536 | A451 | R389 | Y39 | T336 | R242 |
| D474 | D72 | N534 | R450 | Y444 | Y61 | G335 | K190 |
| C475 | A79 | E550 | R447 | H445 | E80 | H334 | V192 |
| D476 | E80 | N496 | E391 | M446 | Q91 | N332 | - |
| E477 | R93 | N482 | E392 | N447 | R93 | N277 | - |
| D478 | L98 | N479 | E394 | D474 | L98 | P276 | - |
| R480 | K100 | P478 | - | N471 | K100 | G275 | - |
| T482 | - | V476 | - | A479 | - | V274 | - |
| D488 | - | E472 | - | R480 | - | L273 | - |
| V491 | - | S466 | - | T482 | - | T266 | - |
| - | - | L464 | - | - | - | L265 | - |
| - | - | - | - | - | - | S264 | - |
| - | - | - | - | - | - | R261 | - |

**Supplementary Table S13.** PCR primer sequences used in this study.

| Primer Name | Sequence (5’-3’) | Uasge |
| --- | --- | --- |
| amhy-F | ACGTGGACAGCAACTGA | Sex identification of three species of Siniperca |
| amhy-R | GAGAAAAGCAGAAGTGG |  |
| M3-F | TGCGCACACATTTAGAGAGG | Sex identification of C. whiteheadi |
| M3-R | GATGGAGCCCCATAGTTTCA |  |
| amhy-orf-F | CATCCCCCTGACTGCGAAA | ORF cloning of amhy |
| amhy-orf-R | CCTCAAACCTGGTGACCTT |  |
| amh-5'RACE-R1 | GCTCGTTGTGTGGTGGTCTC | RACE |
| amh-5'RACE-R2 | TGGTGGTCTCCTGTCATTGTGG |  |
| amh-3'RACE-F1 | AGTGAGAATGTGGACGAG | RACE |
| amh-3'RACE-F2 | TATGAAGCCCTGGAG |  |
| amhy-5'RACE-R1 | GAGGAGGTCAGTGAAGACAT | RACE |
| amhy-5'RACE-R2 | GGTCAGTTGCTGTCCATGTAG |  |
| amhy-3'RACE-F1 | GTGCCTGTGTCCTATGATGTT | RACE |
| amhy-3'RACE-F2 | AAAGGAGTGTGGATGCCGC |  |
| Amhy-gRNA-F | TAATACGACTCACTATAGGACGAGGCTCTGTGTGGCCGGTTTTAGAGCTAGAAATAGC | In vitro transcription of gRNA |
| gRNA-R | AGCACCGACTCGGTGCCACT |  |
| amhy-valid-F | CGTAACTGGTGTCTGCGAG | Validation of amhy mutation |
| amhy-valid-R | TGGAGGCTAATGAGGTCTTT |  |
| β-actin-RT-F | ACAGGGAGAAGATGACCCAGAT | qPCR |
| β-actin-RT-R | CACCGGAGTCCATGACGATA |  |
| QP-dmrt1-F | AGGTGATGGTGAAGAATGAA | qPCR |
| QP-dmrt1-R | CGTGAAGGCTGGTAGAAG |  |
| QP-amh-F | CAGATACATCATGGAGAGTTGGC | qPCR |
| QP-amh-R | TCAGCAGAAGAAATGCACGGTA |  |
| QP-amhy-F | GTGGCCGTGGATGTCCTACA | qPCR |
| QP-amhy-R | GAAGTGGGGTCATGCCGAT |  |
| QP-foxl2-F | GACCTCCACCGACGCACTTC | qPCR |
| QP-foxl2-R | TCACGTTCACCGGACTCACG |  |
| QP-cyp19a1a-F | TCCATGTCCCAGAATGCCAC | qPCR |
| QP-cyp19a1a-R | TGACAGAAGTGGCCCCAAAC |  |
| QP-gsdf-F | GGAGGAGCACCTTCAGCA | qPCR |
| QP-gsdf-R | GCAGGATGGATCACCCAGT |  |
| p4-amh-F | CAGTGTGGTGGAATTATGTTGGTTGTGGAAGTCGTCTACT | Overexpression and transfection |
| p4-amh-R | CGAAGGGCCCTCTAGCACGGCATCCGCACTCC |  |
| p4-amhy-F | CAGTGTGGTGGAATTATGTTTGTTTTGGATGTGTTCTACTGTGG | Overexpression and transfection |
| p4-amhy-R | CGAAGGGCCCTCTAGcGCGGCATCCACACTCCT |  |
| p4-smad4a-F | CAGTGTGGTGGAATTATGTCAATCACGAACACTCCCACA | Overexpression and transfection |
| p4-smad4a-R | CGAAGGGCCCTCTAGaATCAAGGGGCTGAGGGTCA |  |
| p4-smad4b-F | CAGTGTGGTGGAATTATGTCCATCACCAACACACCTACC | Overexpression and transfection |
| p4-smad4b-R | CGAAGGGCCCTCTAGaGTCCAGAGGTTGTGGGTCTG |  |
| p4-smad4c-F | CAGTGTGGTGGAATTATGTGGACTGATTCAGAGCTGGG | Overexpression and transfection |
| p4-smad4c-R | CGAAGGGCCCTCTAGaGTTGGCAGGCCCTGGG |  |
| p4-smad-4dF | CAGTGTGGTGGAATTATGTCGGTGCTCGGCC | Overexpression and transfection |
| p4-smad4d-R | CGAAGGGCCCTCTAGaGAGTGCGTGTTCTCGCGG |  |
| p4-smad1-F | CAGTGTGGTGGAATTATGAACGTCACCTCGCTCTTCT | Overexpression and transfection |
| p4-smad1-R | CGAAGGGCCCTCTAGaGGAGACAGAGGATATAGGGTTGTGG |  |
| p4-smad5-F | CAGTGTGGTGGAATTATGACCTCCATGTCCAGTCTCT | Overexpression and transfection |
| p4-smad5-R | CGAAGGGCCCTCTAGaGGACACAGAGGAGATTGGGTTGAG |  |
| p4-smad8-F | CAGTGTGGTGGAATTATGAACTCCTCTACCTCCATTACGT | Overexpression and transfection |
| p4-smad8-R | CGAAGGGCCCTCTAGaTGACACTGAAGAAATAGGGTTGTGCG |  |
| p4-amhrII-F | CAGTGTGGTGGAATTATGATTCTGCAACAGTGGCGAC | Overexpression and transfection |
| p4-amhrII-R | CGAAGGGCCCTCTAGcTGGAGAGTAACAAGACTGAAGAGAGACTAATCTG |  |
| p4-foxl2-F | CAGTGTGGTGGAATTATGATGGCCACTTACCAAAACGC | Overexpression and transfection |
| p4-foxl2-R | CGAAGGGCCCTCTAGCAATATCAATCCTCGTGTGTAACGCC |  |
| p4-sf1-F | CAGTGTGGTGGAATTATGCATGTGTGCGTTGGTCC | Overexpression and transfection |
| p4-sf1-R | CGAAGGGCCCTCTAGcCACACACGCTCGCTTGG |  |
| pgl-cyp19a1a-pF | GCTCGCTAGCCTCGACAGTCTTTCTCTTGTCTCTCATTGACTCA | Transfection |
| pgl-cyp19a1a-pR | CCGGATTGCCAAGCTCACAAACCCAGCGACCGT |  |
