# Supplementary material for: Assembly and analysis of Sinipercidae fish sex chromosomes reveals that a supergene drives sex chromosome origin and turnover
Source: Adv Biotechnol (Singap). 2025 May 28;3(2):17. doi: 10.1007/s44307-025-00068-6 (PMC12119445; doi:10.1007/s44307-025-00068-6)
Supplement: Supplementary file 5 — Supplementary Material 5. [file 44307_2025_68_MOESM5_ESM.docx]

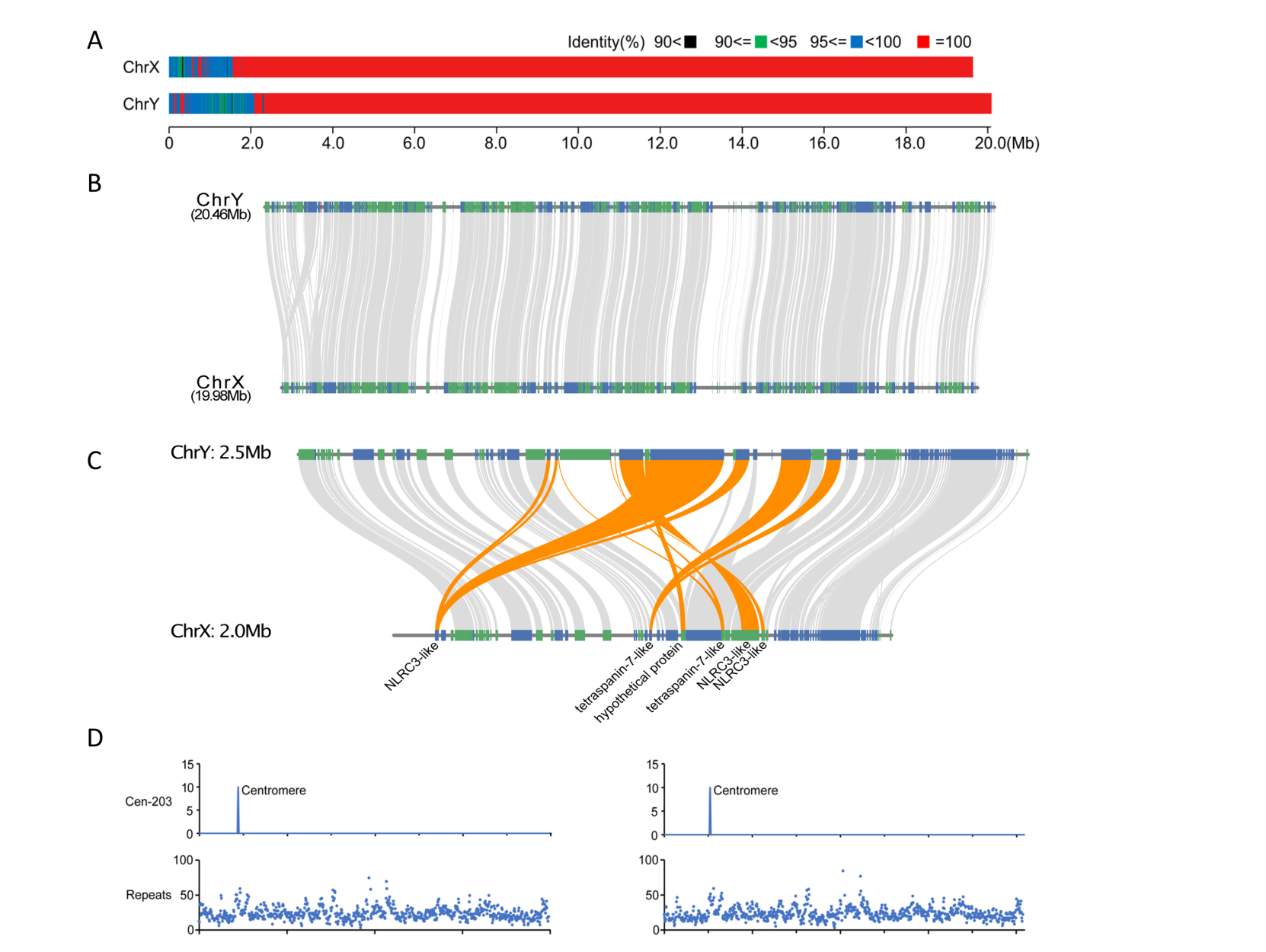


**Supplemental Figure S1.** Characteristics of *S. chuatsi* sex chromosomes. (A) Sequence identity of X and Y chromosomes. (B) Synteny relationships of genes between X and Y chromosomes (Blue represents the genes encoded by the positive strand, green represents the genes encoded by the negative strand and red represents the amhy). (C) Synteny and gene inversion (orange) analysis of the X and Y-link regions. (D) The repeats and location of centromeres based on cen-203 of X and Y chromosomes.


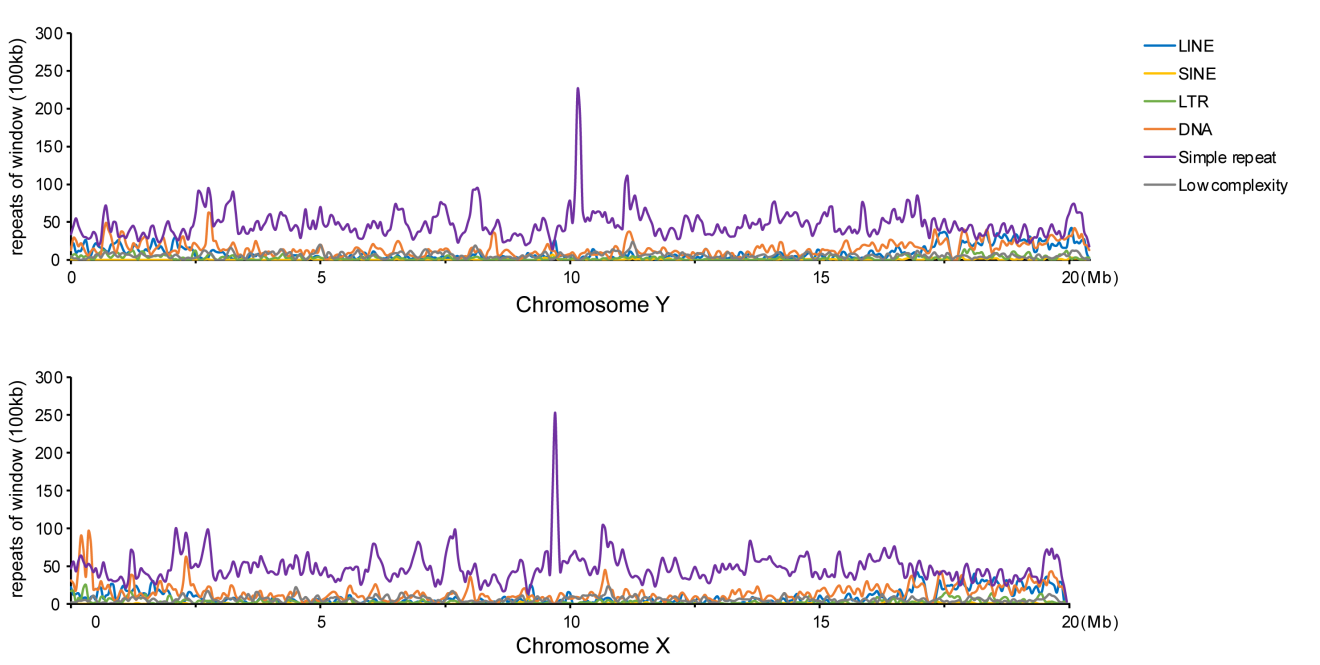


**Supplemental Figure S2.** The distribution of different types of repetitive sequences in X and Y chromosomes.


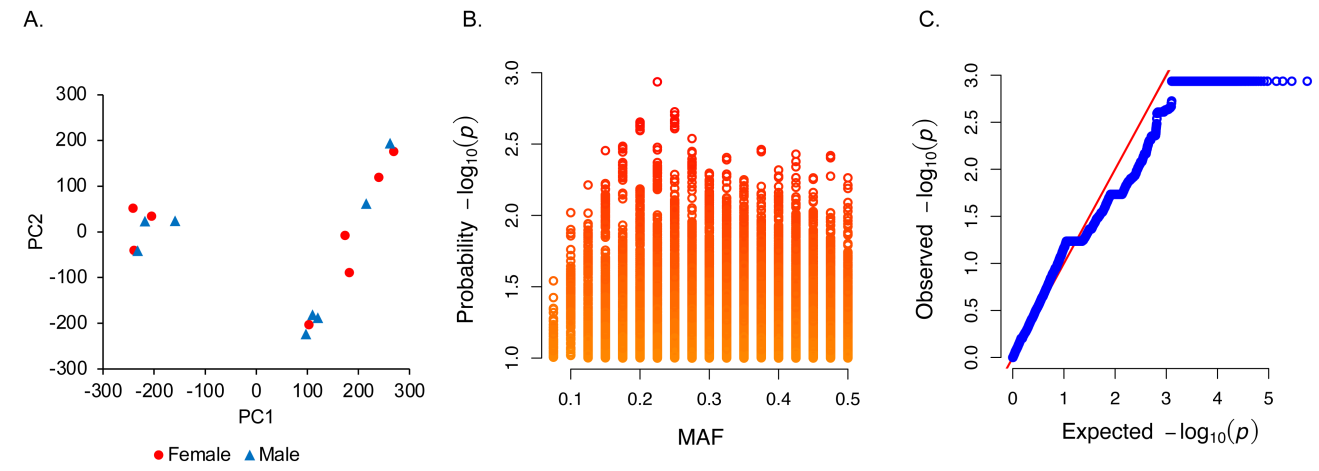


**Supplemental Figure S3.** Quality control measures applied to the GWAS results. (A) The PCA analysis of samples based on SNP sites, indicating that the source population of the test species is not a single population (at least two populations, divided into two clusters along the PC1 axis). (B) The relationship between MAF (Minor Allele Frequency) and logP. For SNPs associated with sex, the corresponding MAF is 0.25, and the -logP value is the highest. (C) The QQ-plot based on the GLM model. The topmost points on the Observed -logP axis (Y-axis) correspond to sex-specific SNPs.

**
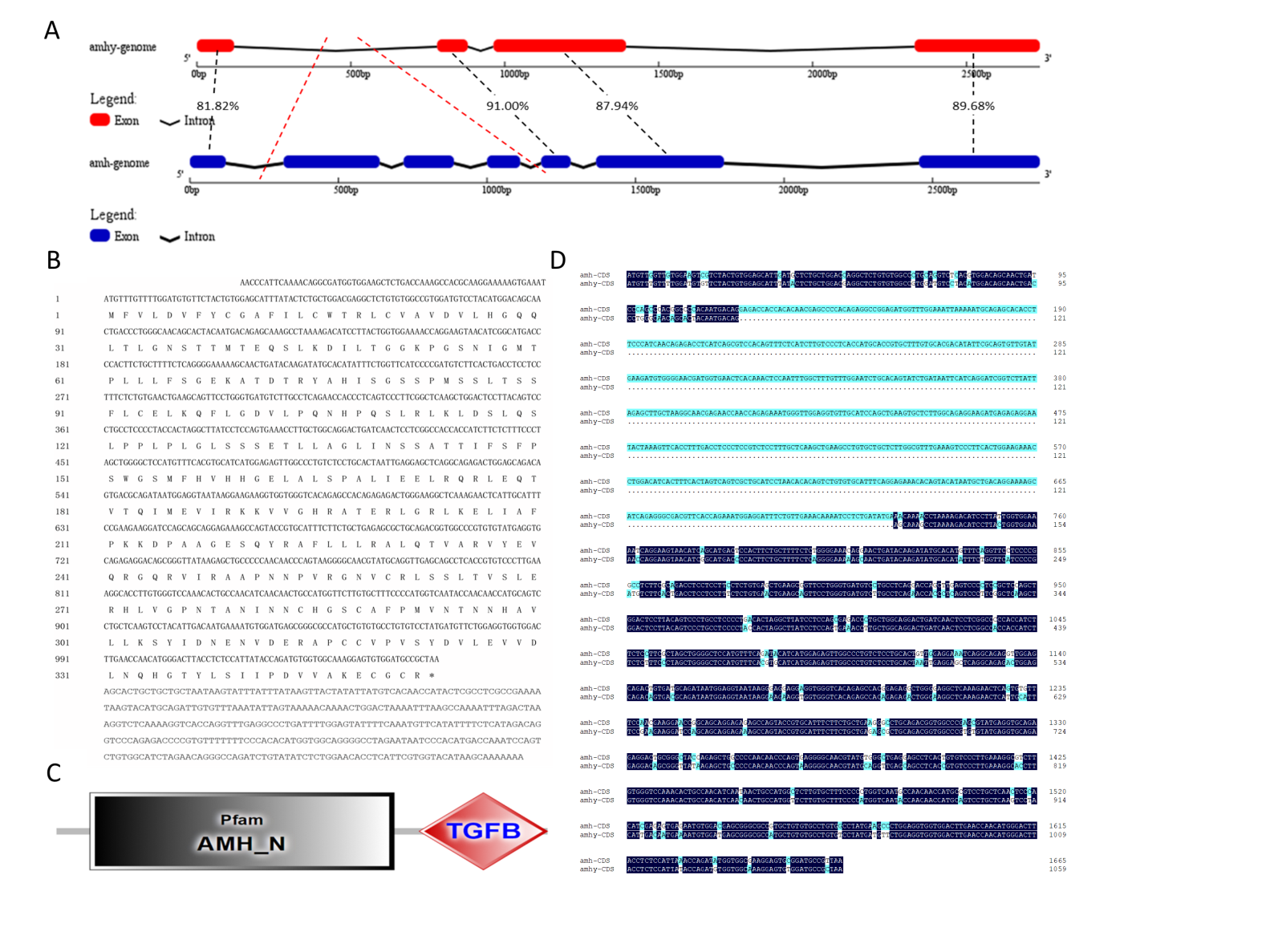
**

**Supplemental Figure S4.** Sequence characterization of *amhy* in *S. chuatsi*. (A) Comparison of genomic sequence structure of *amhy* and *amh*. (B) The complete mRNA sequence of *amhy*. (C) The domain composition of *amhy*. (D) The alignments of protein coding sequences (CDS) of *amh* and *amhy*. Black blocks indicate 100% similarity.


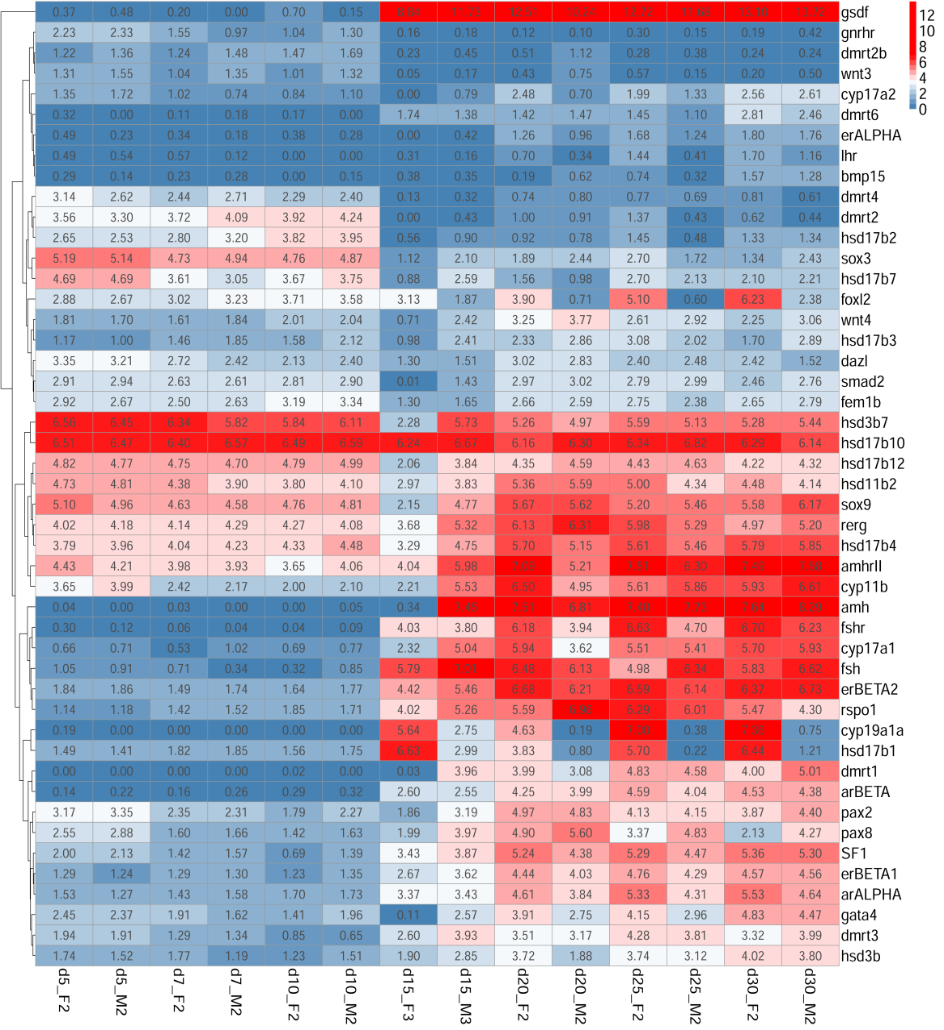


**Supplemental Figure S5.** Heat map showing the gene expression of sex determination genes during early gonad development based on transcriptomic analysis.


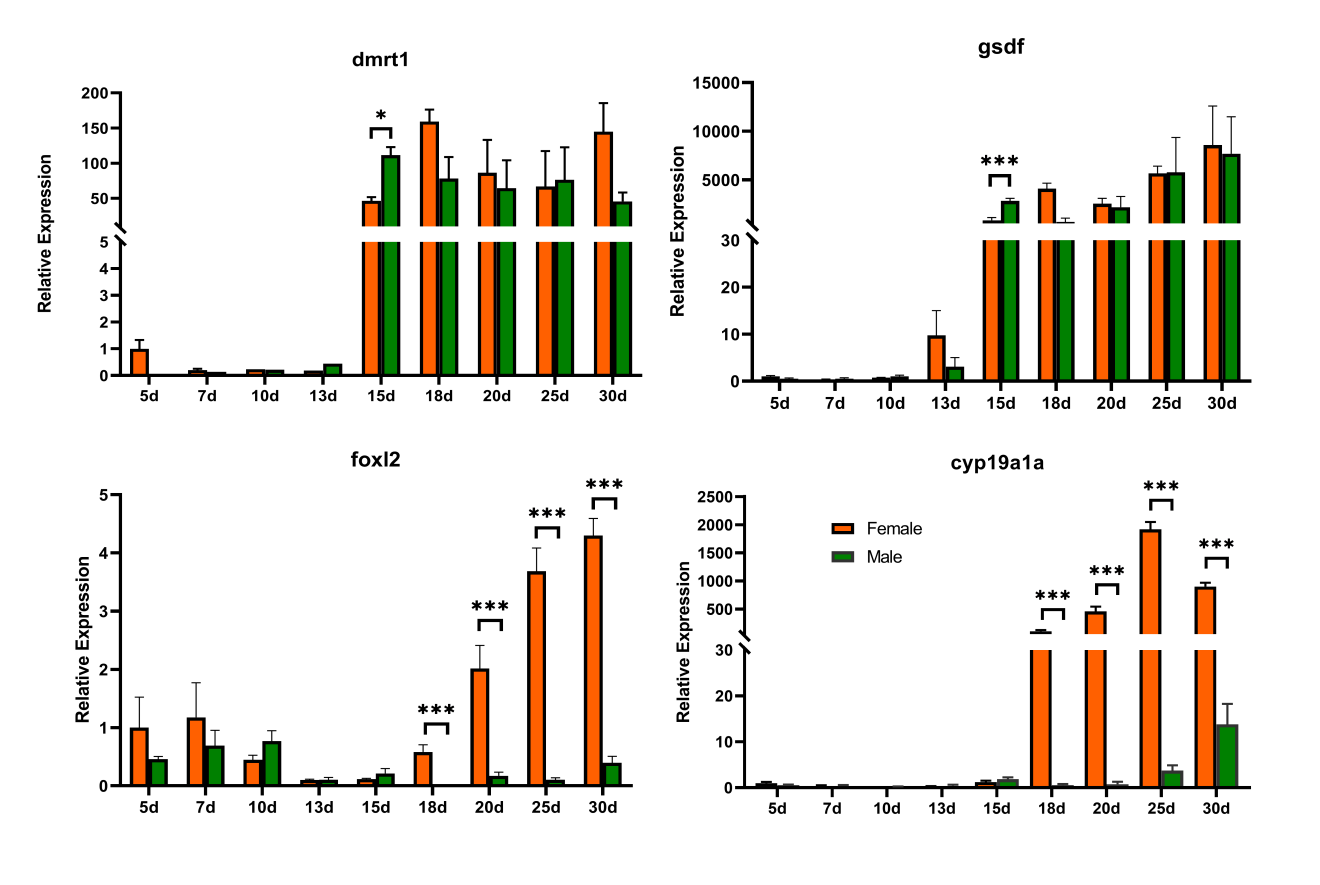


**Supplemental Figure S6.** Expression analysis of key genes involved in sex determination and differentiation in early gonads based on RT qPCR.


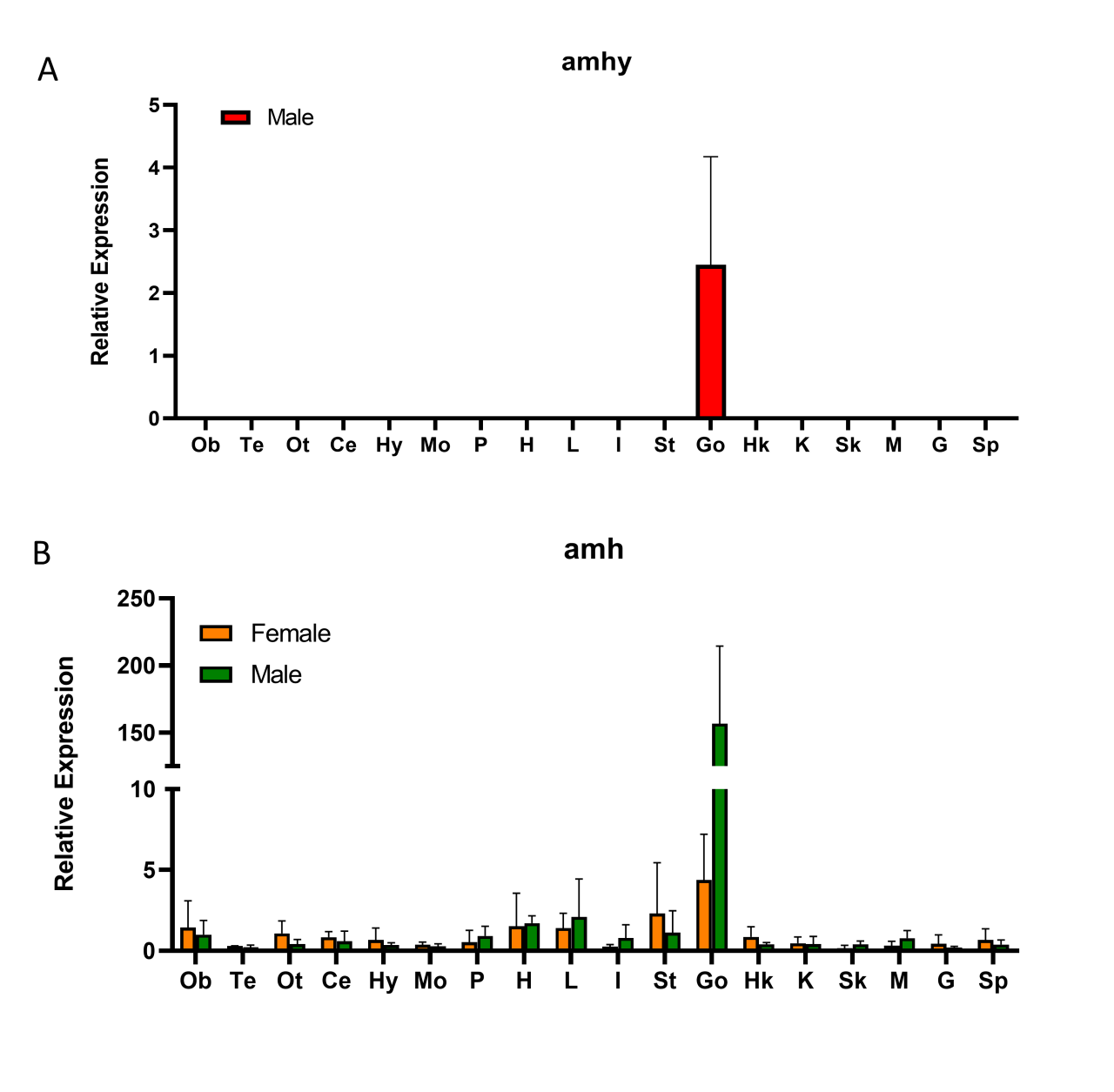


**Supplemental Figure S7.** Tissue-specifc expression of *amh* and *amhy* based on RT qPCR. Ob, olfactory bulb; Te, telencephalon; Ot, optic-tectum; Ce, cerebellum; Hy, hypothalamus; Mo, medulla oblongata; P, pituitary; H, heart; L, liver; I, intestine; St, stomach; Go, Gonad; Hk, head kidney; K, kidney; Sk, skin; M, muscle; G, gill; Sp, spleen.

**Supplemental Figure S8.** Sequence alignment of *amh* and *amhy* promoters (3,000 bp upstream of ATG) in mandarin fish. Black blocks indicate 100% similarity.


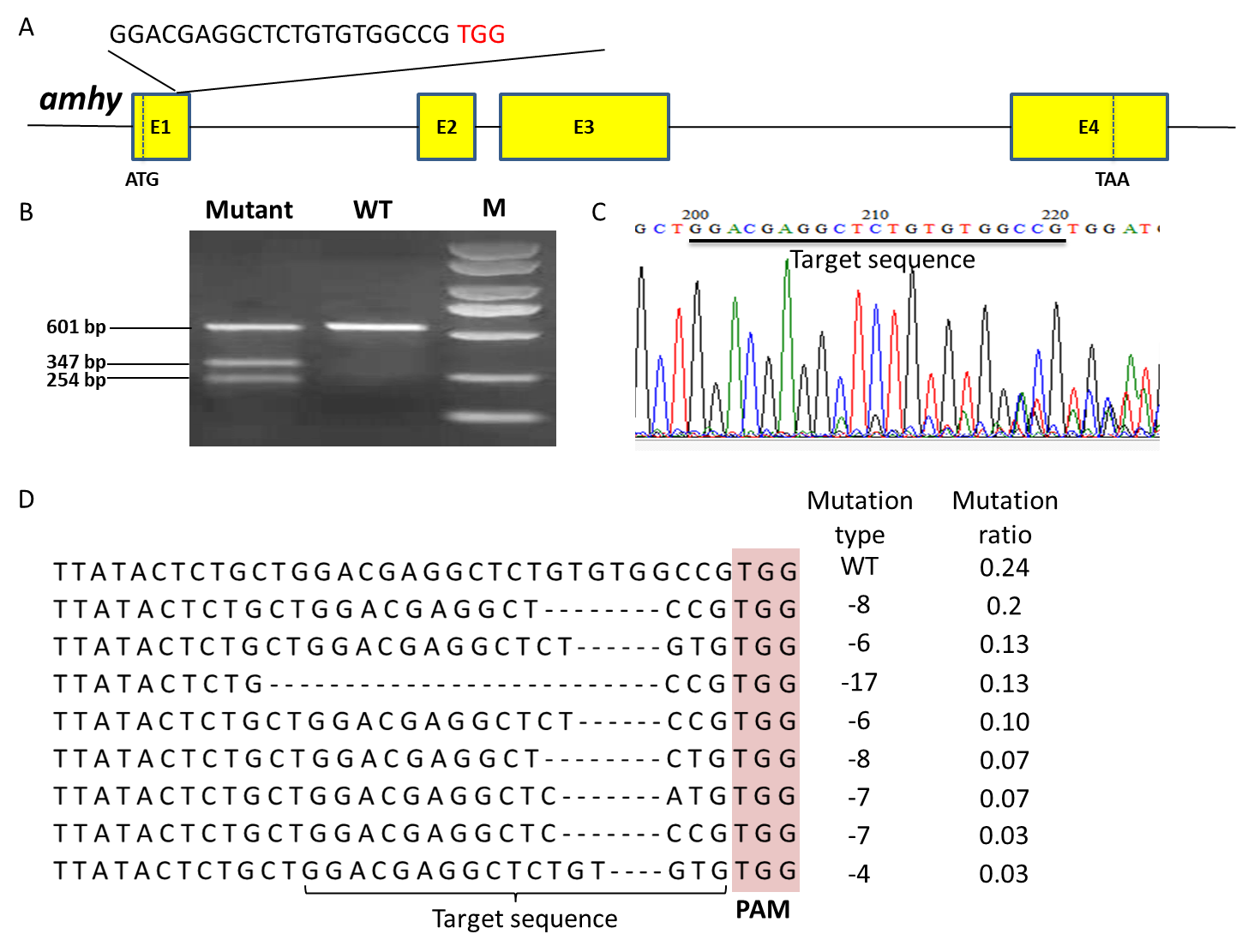


**Supplemental Figure S9.** CRISPR/Cas9 mutation target selection and mutation detection of *amhy* genes. (A) The CRISPR/Cas9 target site of *amhy*. (B) Mutation detection using T7 Endonuclease I. (C) Mutation confirmation based on Sanger sequencing. (D) Mutation type and rate detection in the F0 generation.

**
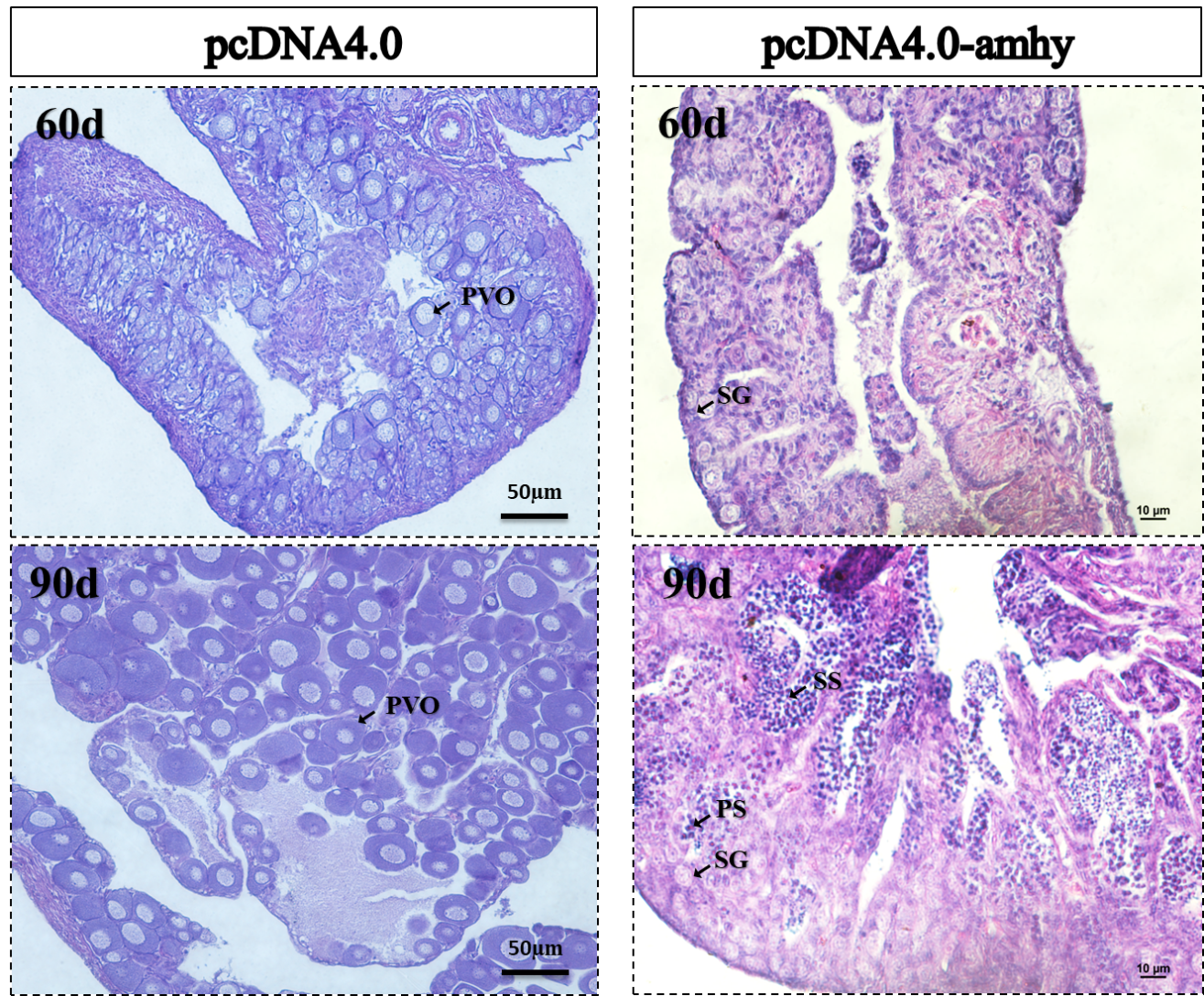
**

**Supplemental Figure S10.** Gonad histology section for the *amhy* overexpression group (pcDNA4.0-Amhy) and control group (pcDNA4.0). PVO, pre-vitellogenic oocytes; SG: spermatogonium; PS: primary spermatocyte.


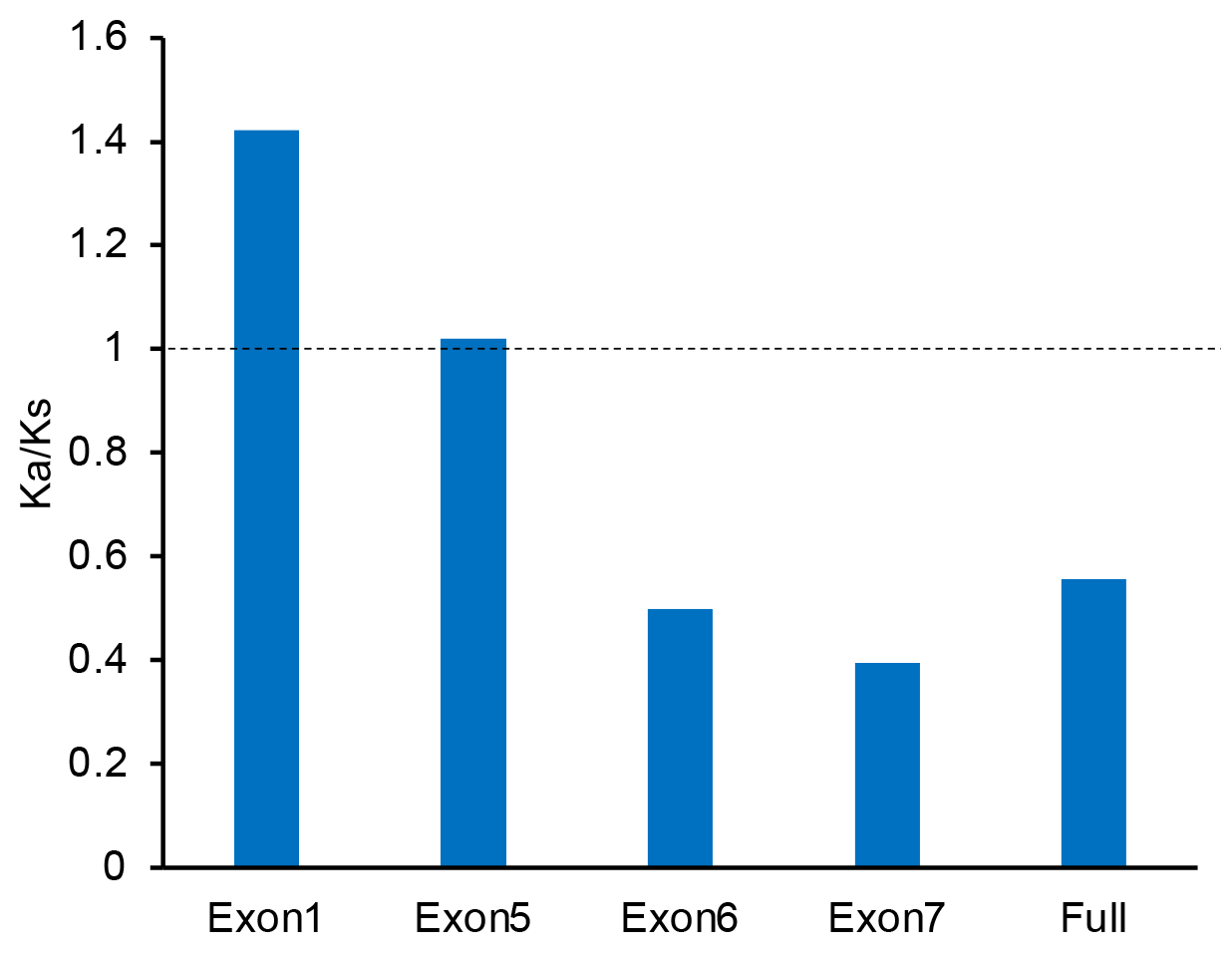


**Supplemental Figure S11.** Ka/Ks values of *amhy/amh* for the full genes and for each exon.

**
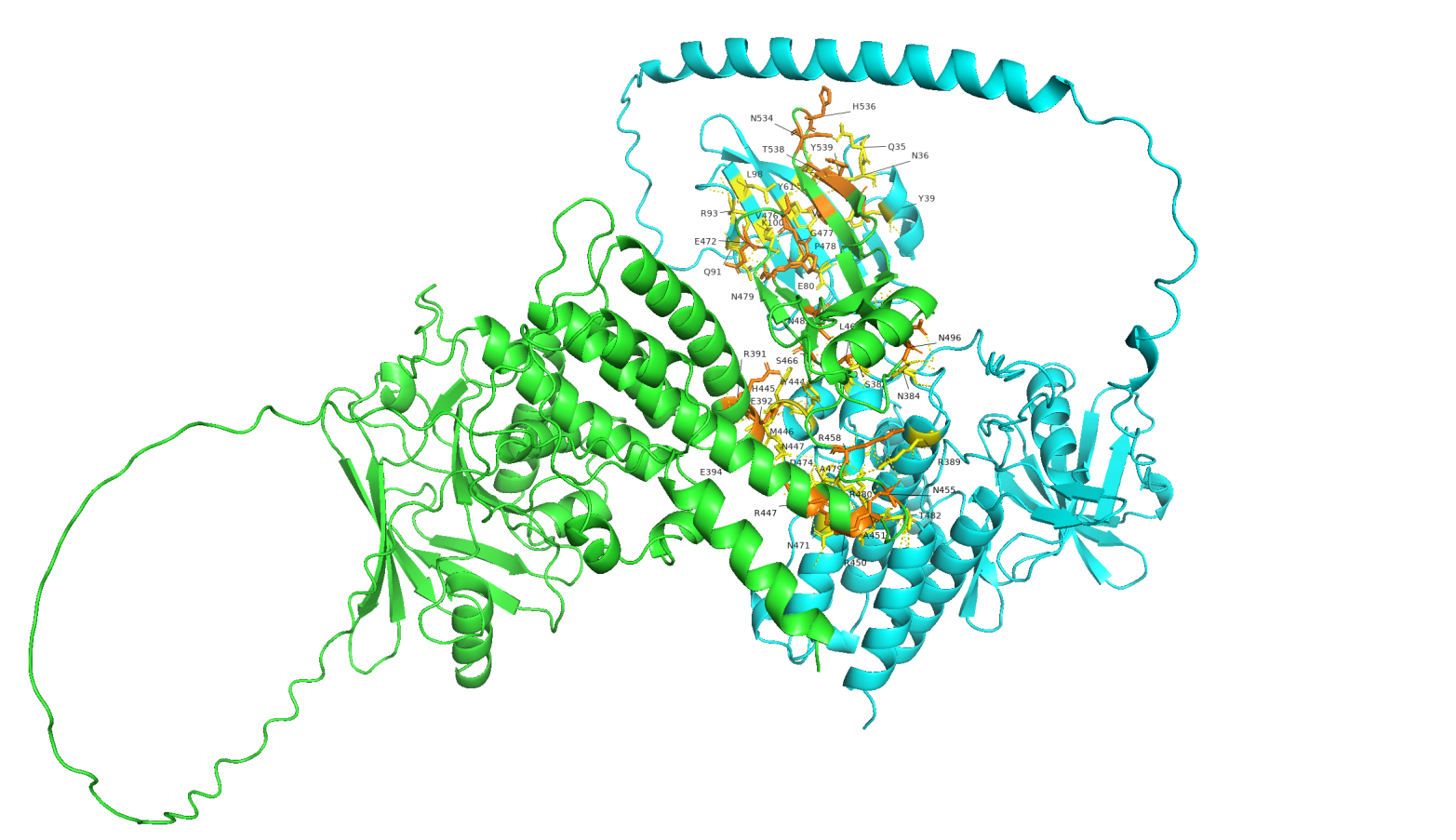
**

**Supplemental Figure S12.** The predicted protein interaction model between AMH (green) and AMHRII (blue) by alphafold 3 (ipTM=0.35; pTM=0.43) (Yellow color indicates the interaction sites on AMHRII and orange color indicates the interaction sites on AMH).

**
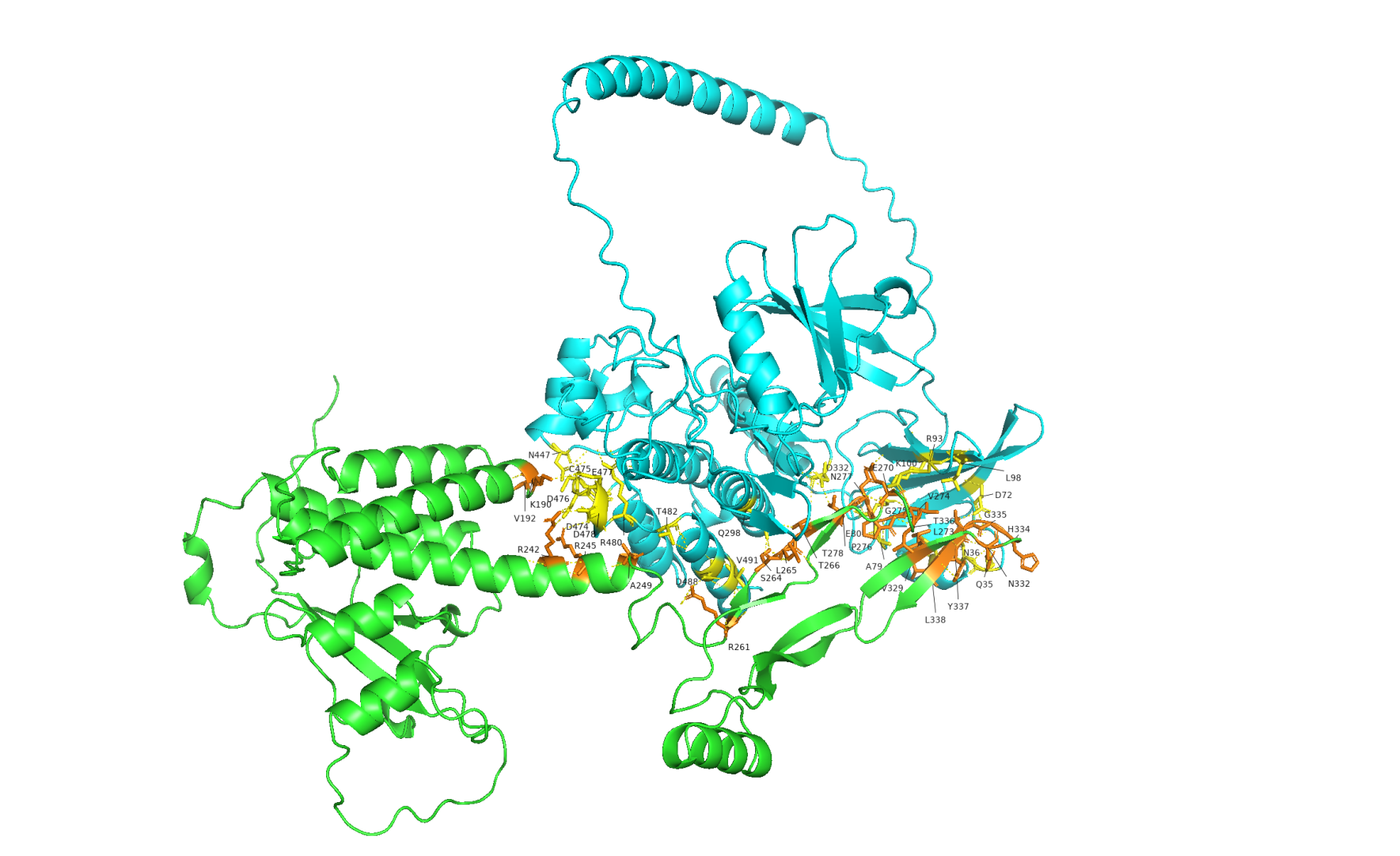
**

**Supplemental Figure S13.** The predicted protein interaction model between AMHY (green) and AMHRII (blue) by alphafold 3 (ipTM=0.36; pTM=0.44) (Yellow color indicates the interaction sites on AMHRII and orange color indicates the interaction sites on AMHY).

**
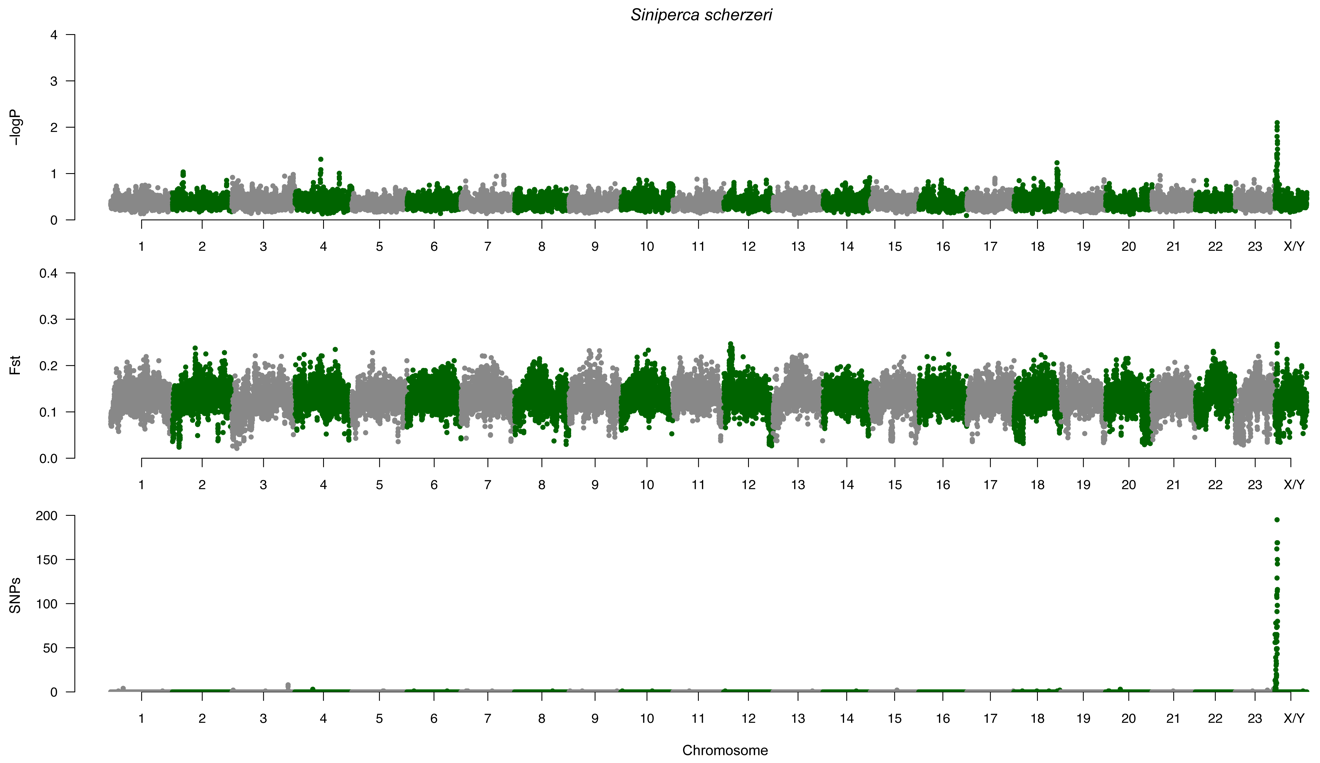
**

**Supplemental Figure S14.** Manhattan plot showing relationships among sex related SNPs, -logP, and Fst analysis for the whole genome of *S. scherzeri*.

**
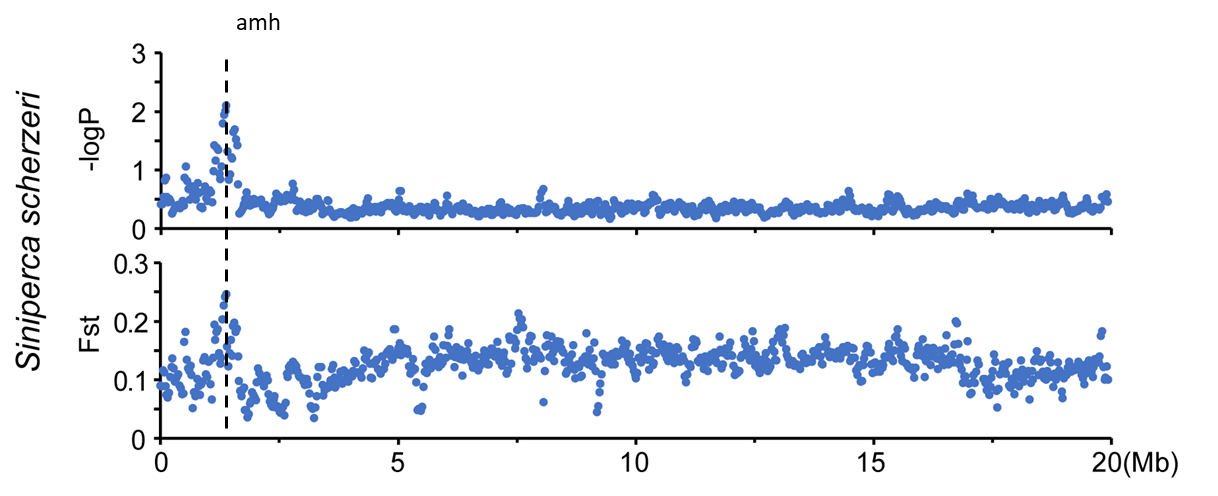
**

**Supplemental Figure S15.** Manhattan plot showing relationships among the -logP of sex related SNPs and Fst values of sex chromosome of *S. scherzeri*. The Y specific *amh* location is indicated by a vertical dotted line.

**
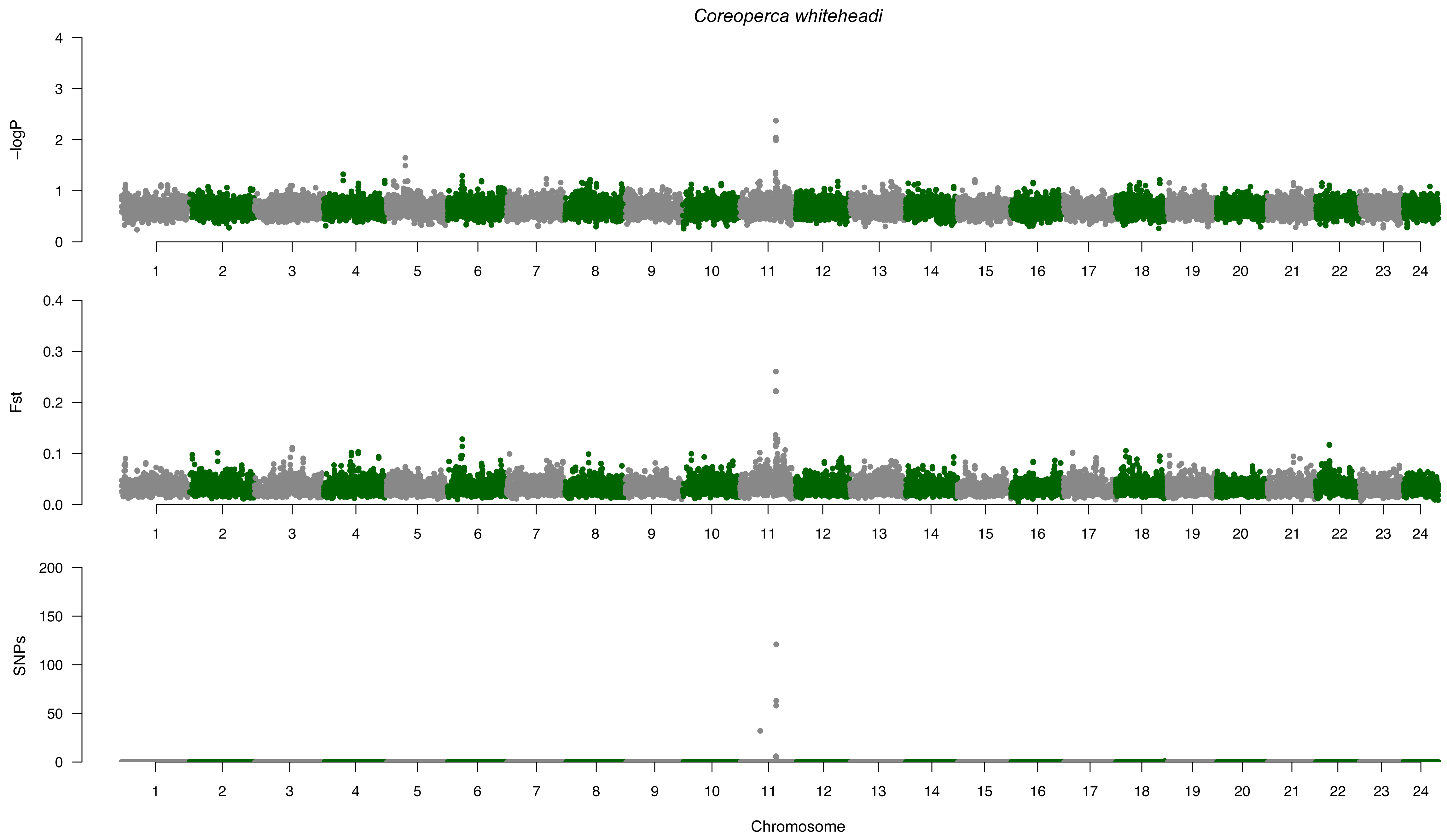
**

**Supplemental Figure S16.** Manhattan plot showing the association of sex related SNPs, -logP, and Fst values for the whole genome of *C. whiteheadi*.

**
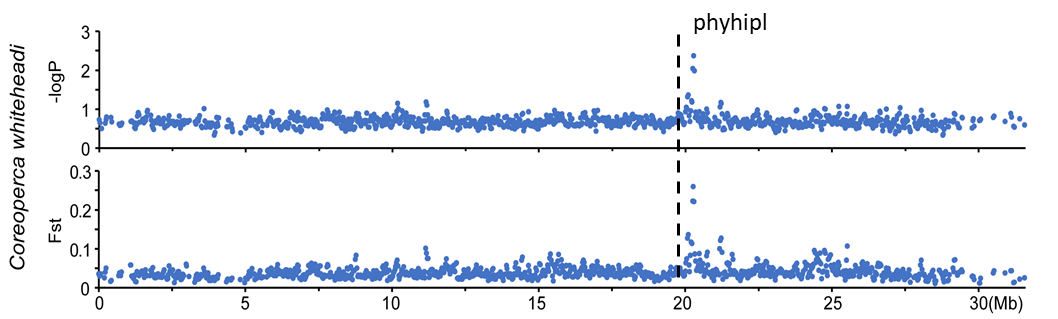
**

**Supplemental Figure S17.** Manhattan plot showing the associations of –logP of sex related SNPs and Fst values for the sex chromosome of *C. whiteheadi*. The *phyhipl* location is indicated by a vertical dotted line.

**
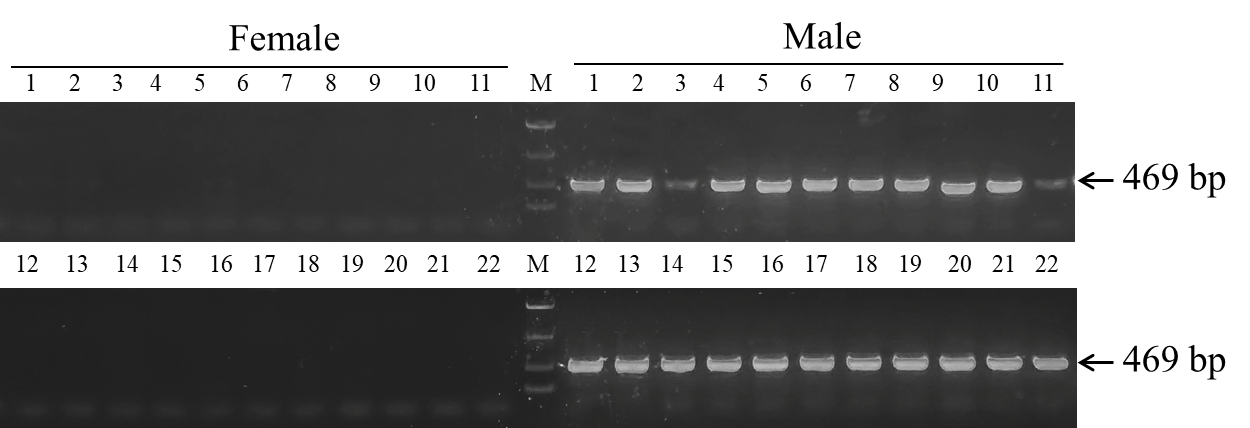
**

**Supplemental Figure S18.** PCR detection and genetic sexing using the M3 marker for 22 females and 22 males of *C. whiteheadi*.

**Supplemental Figure S19.** The alignment of *amhy* sequences (containing CDS and intron) in *Siniperca* fishes including *S. chuatsi* (SC), *S. scherzeri* (SS) and *S. knerii* (SK). The intron is underlined.

**Supplemental Figure S20.** The alignment of promoter sequences of *amhy* in *Siniperca* fishes including *S. chuatsi* (SC), *S. scherzeri* (SS) and *S. knerii* (SK).

**
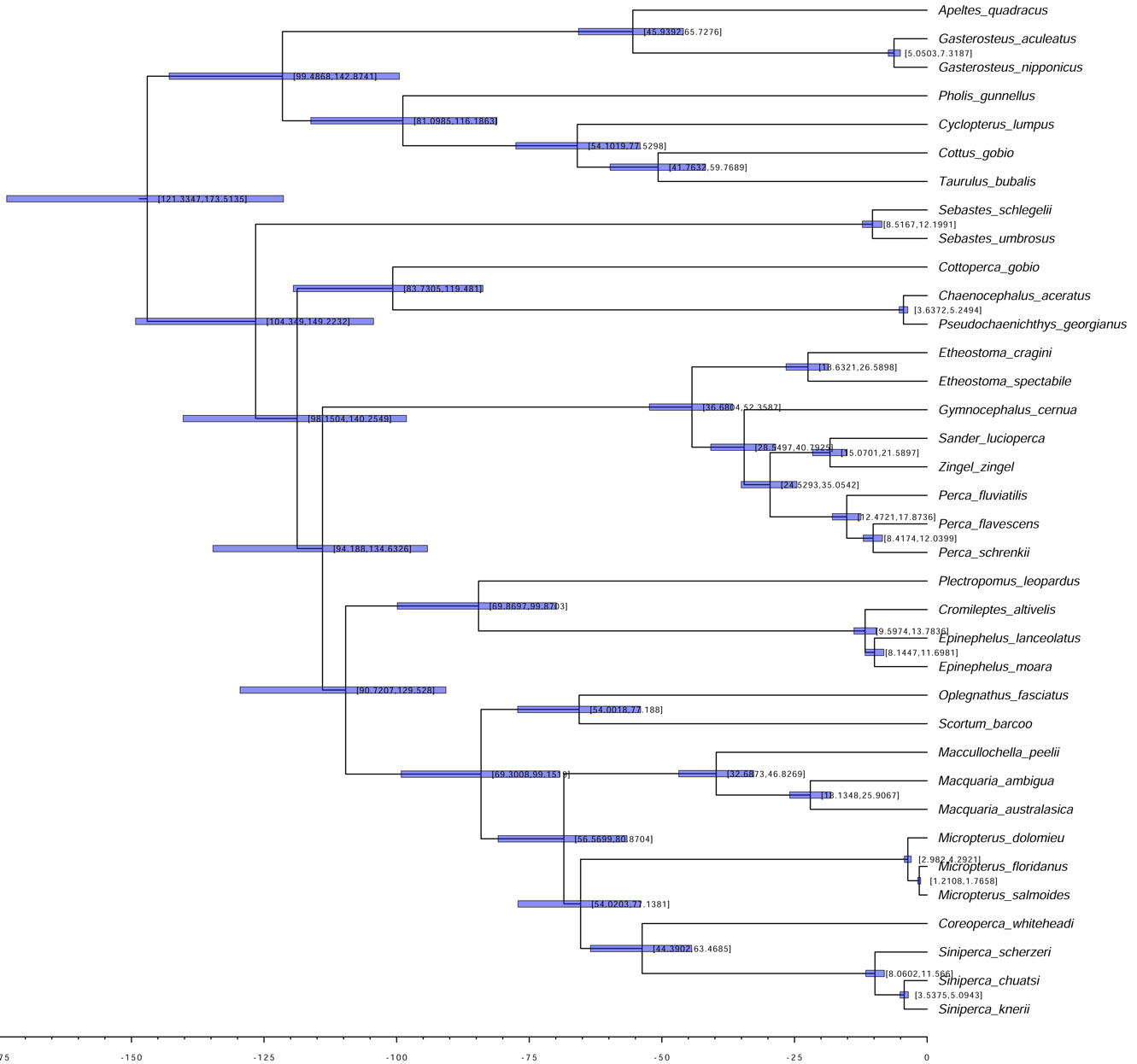
**

**Supplemental Figure S21.** Timetree calculated from single copy orthologous genes from 36 Perciformes fish species.

**Supplemental Figure S22.** Sequence alignment of *amhx*, the genome sequences, and protein coding sequences of *amh*.


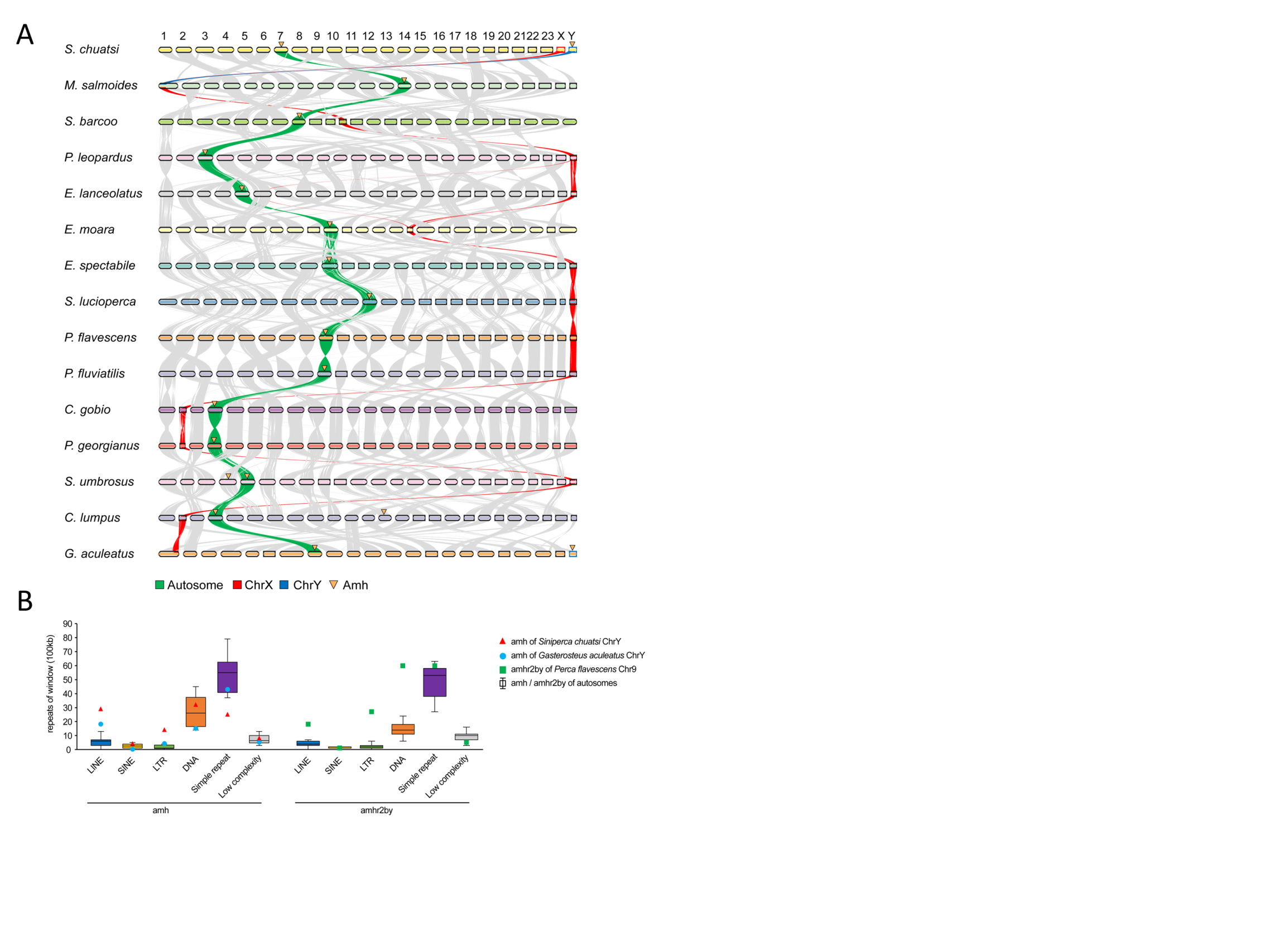


**Supplemental Figure S23.** **Collinearity and repeat element analysis.** (A) Chromosome collinearity analysis of several Perciformes species. (B) The divergence of different repetitive elements in corresponding regions harboring *amh* or *amhr2* between sex chromosomes and autosomes.
